# Extract of *Curculigo capitulata* Ameliorates Postmenopausal Osteoporosis by Promoting Osteoblast Proliferation and Differentiation

**DOI:** 10.3390/cells13232028

**Published:** 2024-12-08

**Authors:** Ying Wang, Xueru Wang, Kaijin Wang, Weiwei Qin, Ning Li

**Affiliations:** Inflammation and Immune Mediated Diseases Laboratory of Anhui Province, School of Pharmacy, Anhui Medical University, Hefei 230032, China; 18326039565@163.com (Y.W.); 19855867686@139.com (X.W.); wkjahla@163.com (K.W.)

**Keywords:** *Curculigo capitulata*, ERα, osteoblast proliferation and differentiation, PMOP, PI3K/AKT

## Abstract

Postmenopausal osteoporosis (PMOP) is a bone disease characterized by bone thinning and an increased risk of fractures due to estrogen deficiency. Current PMOP therapies often result in adverse side effects. The traditional medicinal plant *Curculigo capitulata* is commonly used to strengthen bones and support kidney function, but its role in treating PMOP is not well understood. This study aims to investigate the therapeutic effects of the total extract of *Curculigo capitulata* (Eocc) on PMOP and to explore the underlying mechanisms. The major components of the extract were identified using HPLC. Transcriptomics was employed to predict potential targets. An osteogenic differentiation model of MC3T3-E1 cells was used in vitro. The osteogenic potential of the Eocc was assessed through CCK-8 cell viability assays, alkaline phosphatase (ALP) staining, Alizarin Red staining, Western blotting, and qPCR. MCF-7 and HEK-293 cells were utilized to evaluate the estrogen-like activity of Eocc. Apoptosis rates were detected by flow cytometry. In vivo, a bilateral ovariectomized mouse model of PMOP was used to further validate the in vitro findings through histopathological analysis and WB results. The results demonstrated that the Eocc promoted the proliferation of MC3T3-E1 cells, increased ALP activity, and stimulated the formation of osteogenic mineralized nodules. It also upregulated the expression of osteogenic markers (Runx2, OCN, OPN, and BSP) at both the protein and mRNA levels. The Eocc induced the activation of ERα both in vitro and in vivo, initiating the Src/PI3K/AKT signaling pathway, leading to the phosphorylation of GSK3β and subsequent osteogenesis. The activation of this pathway also stimulated the phosphorylation of mTOR and p70S6K while downregulating cleaved caspase-3 and caspase-9. Additionally, the Eocc reduced apoptosis during osteogenic differentiation and promoted cell proliferation. These findings suggest that the Eocc facilitates osteoblast proliferation and differentiation, improving bone integrity in PMOP mice, and may represent a promising therapeutic candidate for managing PMOP.

## 1. Introduction

### 1.1. Osteoporosis (OP) and Postmenopausal Osteoporosis (PMOP)

Osteoporosis (OP) is a degenerative bone disease caused by an imbalance in the bone remodeling process [1]. Type I osteoporosis, also known as postmenopausal osteoporosis (PMOP) or estrogen-deficient osteoporosis, commonly occurs in women after menopause [2]. The primary cause of PMOP is the reduction in endogenous estrogen, which leads to accelerated bone loss in postmenopausal women [3]. As a result, approximately 70% of PMOP patients experience fractures in the spine, wrist, and hip, which can lead to a loss of mobility and independence and even result in death [4].

### 1.2. Treatment Status and Disease Mechanism of PMOP

Current preventive treatments for PMOP, including bisphosphonates, denosumab, parathyroid hormone (PTH) analogs, and hormone replacement therapy (HRT), are effective to some extent. However, these treatments often come with significant adverse effects, such as an increased risk of cancer and cardiovascular diseases [3,5,6,7]. Given the limitations and potential health risks of these therapies, there is an urgent need to develop more effective and safer therapeutic compounds for the treatment of PMOP.

The fundamental principle of PMOP is the imbalance between osteoblast-mediated bone formation and osteoclast-mediated bone resorption [8,9]. This age-related osteoporosis is often associated with osteoblast dysfunction, and impaired osteoblast activity leads to reduced bone formation, disrupting the dynamic balance of bone remodeling [10]. The first-line treatment drug teriparatide also treats PMOP by inducing osteoblast differentiation, stimulating existing osteoblasts to form new bone, and reducing osteoblast apoptosis [11]. In addition, the key factors in PMOP, estrogen and its receptors, play a significant cell-specific role in the development and maintenance of bone mass. Estrogen inhibits osteoblast apoptosis and induces osteoblast differentiation [12]. Therefore, discovering new therapeutic approaches to promote osteoblast differentiation is of great importance for the prevention and treatment of PMOP.

### 1.3. Potential Role of Curculigo Capitulata in the Treatment of PMOP

Traditional Chinese medicine (TCM) has a history spanning thousands of years in the prevention and treatment of osteoporosis. The use of bioactive compounds extracted from TCM has shown fewer side effects and greater sustainability [13]. According to records, the plants of the genus *Curculigo* are frequently used to tonify the kidneys, enhance yang, and strengthen bones [14]. Chlorophenolic glycosides and polysaccharides found in genus *Curculigo* have shown a proliferative effect on osteoblasts in vitro [15,16,17]. Characteristic phenolic compounds in genus *Curculigo*, such as curculigoside and orcinol glucoside, have also been shown to treat osteoporosis in vivo and in vitro through signaling pathways such as Wnt/β-catenin, Akt/FoxO1, Nrf2/Keap1, and mTOR [18,19,20,21]. Therefore, we hypothesize that *Curculigo capitulata* (Lour) O. Ktze, a member of the genus *Curculigo*, is also a potential anti-osteoporosis drug. However, there are currently no studies that have demonstrated its related effects.

It is well known that estrogen activity is primarily mediated by two nuclear receptors, estrogen receptor α (ERα) and estrogen receptor β (ERβ) [22]. Studies have shown that ERα appears to be the main mediator of estrogen’s actions on the skeleton [23]. Single nucleotide polymorphisms of ERα can affect bone mineral density (BMD), rates of bone loss, and fracture risks in both men and women [24]. This may be related to the osteoprotective effects of estrogen in PMOP.

Therefore, there are currently many studies focusing on the ability of natural products to activate ERα for the treatment of PMOP. Natural products can promote osteoblast differentiation by activating the ERα and Wnt/β-catenin signaling pathways [25]. They can also inhibit RANKL-induced osteoclastogenesis through the ERα/miR-503/RANK signaling pathway in RAW264.7 cells [26]. The PI3K/AKT signaling pathway is also closely associated with osteoporosis. Natural products can stimulate osteoblastic bone formation by activating the PI3K/AKT signaling pathway, enhance the differentiation of bone marrow mesenchymal stem cells into osteoblasts, and inhibit osteoblast apoptosis [27,28,29,30]. Additionally, they can suppress osteoclastogenesis and reduce bone loss through the PI3K-AKT-NFATc1 pathway [31].

In this study, we prepared a total extract of *Curculigo capitulata* (Eocc) and identified its main components using HPLC. We determined the targets of action through a combination of network pharmacology and transcriptomics. The anti-osteoporosis effects and related mechanisms of Eocc were confirmed in PMOP mice and MC3T3-E1 cells. This is the first exploration of the anti-osteoporosis effects of *Curculigo capitulata*, providing a new direction for the treatment of PMOP with this extract as a pioneering approach.

## 2. Materials and Methods

### 2.1. Preparation of the Extract of Curculigo capitulata (Eocc)

The roots and rhizomes of *Curculigo capitulata* (Lour.) Kuntze were collected in Leshan, Sichuan Province, China, in July 2022. The species was authenticated by Prof. Kai-Jin Wang from Anhui University, and a voucher specimen (20220708) was deposited in the School of Pharmacy at Anhui Medical University. The plant name was confirmed at the online database “WFO Plant list” (https://wfoplantlist.org/, accessed on 1 November 2024).

A total of 10 kg of dried rhizomes of *Curculigo capitulata* was placed in a multifunctional extraction and concentration unit and soaked in 75 L of 95% ethanol for 24 h. Then, the extract was performed at 60 °C for 24 h three times. All the filtrates were collected, concentrated under reduced pressure at 45 °C, and then freeze-dried to obtain the Eocc (1350 g). Store the Eocc at 4 °C, and dilute to 0.1 g/mL before use in experiments. The quality of the Eocc was evaluated using high-performance liquid chromatography (HPLC, Thermo Scientific UltiMate 3000, Shanghai, China) with a diode array detector (DAD) at the wavelength of 254 nm for measurement.

### 2.2. Animals and Eocc Administration

Female C57BL/6J mice, 8 weeks old, weighing 18–22 g, were obtained from Hangzhou Ziyuan Laboratory Animal Science and Technology Co., Ltd. (Production Certificate No: SCXK (Zhejiang) 2019-0004; Quality Certificate No: 20240327Abzz0105000561). The animals were housed in individually ventilated cages (IVCs) under controlled conditions of 22 ± 2 °C and 60 ± 5% humidity. All animal experiments were conducted in compliance with ethical standards and received approval from the Ethics Committee of Anhui Medical University (Ethics Approval No: LLSC20220879).

Following a one-week acclimation period, the mice underwent surgery to establish the ovariectomized osteoporosis (OVX-OP) model, while an additional eight mice underwent sham surgery. One week post-surgery, the mice were divided into the following groups, with six mice per group: (1) sham group: the intraperitoneal injection of saline solution; (2) OVX group: the intraperitoneal injection of saline solution; (3) E2 group: the intraperitoneal injection of E2 (1 mg/kg); (4) E-L group: the intraperitoneal injection of the Eocc (10 mg/kg); and (5) E-H group: the intraperitoneal injection of the Eocc (20 mg/kg). All treatments were administered every three days, and body weight changes in the mice were recorded throughout the treatment period. After 6 weeks of treatment, the mice were sacrificed by cervical dislocation under halothane anesthesia, and femurs were collected for further analysis (Table 1).

### 2.3. Micro-Computed Tomography (Micro-CT)

Before decalcification, the collected femurs were fixed in 10% PFA and scanned using Micro CT (NEMO, NMC-200). The scanning parameters were set as follows: tube voltage (90 kV); tube current (40 µA); voxel size (0.03 × 0.03 × 0.03 mm); and scanning resolution (14 µm). Further method selections are as follows: data acquisition: Cruiser; image reconstruction: Recon; and image display and analysis: Avatar. The regions of interest (ROIs) included the trabecular bone and cortical bone. Structural parameters of the trabecular and cortical bones were measured.

### 2.4. Cell Source and Cell Culture

MCF-7 human breast cancer cells (SCSP-531) were obtained from the Cell Resource Center, Institute of Basic Medical Sciences, Chinese Academy of Medical Sciences. HEK-293 human embryonic kidney cells (iCell-h243) were purchased from iCell Bioscience Inc. (Shanghai, China) MC3T3-E1 Subclone 14 cells (STCC20026P) were acquired from Servicebio.

The complete medium (CM) consists of MEM (Product No.: 11095080, Thermofisher, Shanghai, China) with 10% fetal bovine serum, 100 U/mL of penicillin, and 100 µM of streptomycin. The estrogen-deficient medium (EDM) replaces 5% fetal bovine serum with 10% charcoal-stripped fetal bovine serum (Product No.: U712-001, Lonsera). The osteogenic induction medium (OIM) consists of MEMα (Product No.: 11095080, Thermofisher, Shanghai, China) with 10% fetal bovine serum, 100 U/mL of penicillin, and 100 µM of streptomycin, supplemented with 5 mM of β-Glycerophosphate (CAS No.: 13408-09-8, Product No.: HY-D0886, MedChemExpress, Shanghai, China) and 50 µg/mL of ascorbic acid (CAS No.: 50-81-7, Product No.: A103539, Aladdin). In vitro cell culture was performed in a humidified incubator with 5% CO_2_. HEK293 cells and MCF-7 cells were cultured in the CM. MC3T3-E1 cells were cultured in the Specific Culture Medium for MC3T3-E1 Subclone 14 Cells (Product No.: GZ20026-500ML, Servicebio, Wuhan, China).

### 2.5. Experimental Design In Vitro

The in vitro experimental procedures involved the treatment of three cell lines. For the E-SCREEN assay using MCF-7 cells, the normal group was cultured in the CM, while the E2 group (treated with 1 μM of 17β-estradiol) and the Eocc groups (treated with 0.1, 1, or 10 μg/mL of the Eocc) were cultured in the EDM for 24 h. In the Dual Luciferase Reporter Assay with HEK293 cells, all groups were cultured in the CM. The normal and control groups received no treatment, while the E2 group was treated with 1 μM of 17β-estradiol. The Eocc groups (E-1, E-2, E-3) were treated with 0.1, 1, or 10 μg/mL of the Eocc, respectively. The ICI-1 group was treated with 0.1 μg/mL of the Eocc and 1 μM of ICI 182,780, and the ICI-2 group received 1 μM of 17β-estradiol and 1 μM of ICI 182,780. Finally, osteogenesis-related experiments using MC3T3-E1 cells (e.g., cell proliferation, ALP staining, and Alizarin Red staining) included normal, control, E-1, E-2, E-3, and ICI-1 groups, with treatments following the same protocol as in the Dual Luciferase Reporter Assay. The normal group was cultured in the MC3T3-E1-specific medium, while all other groups were cultured in the OIM. Treatment durations varied, with cell proliferation and flow cytometry conducted after 24 h, WB, qPCR, and ALP staining after 7 days (with the medium replaced every 2 days), and Alizarin Red staining after 21 days (with the medium replaced every 3 days). Details are provided in Table 2.

### 2.6. Cell Viability Assay

Cell viability was measured using the CCK-8 kit (Product No.: BMU106-CN, Abbkine) [32]. MC3T3-E1 cells were seeded in 96-well plates (8000 cells per well). Use the specialized culture medium to culture the cells or dilute the drug. After incubation, the cells were treated with different concentrations of the Eocc. After 24 h, the cells were incubated at 37 °C for 2–4 h, and, then, 10 µL of CCK-8 solution was added to each well. Absorbance (OD value) at 450 nm was measured using a microplate reader.

### 2.7. Alkaline phosphatase (ALP) Activity Assay and Mineralization Assay

The normal group was cultured in a specialized culture medium, while the control and treatment groups were induced to osteogenesis using the OIM. The fresh drug-containing induction medium was replaced every 3 days. On the 7th day of induction, the cells were fixed with 4% paraformaldehyde at room temperature for 10 min and stained for alkaline phosphatase using an ALP staining kit (Product No.: CTCC-JD002, Puhebio) according to the manufacturer’s instructions. The staining was observed and photographed under a microscope. After 21 days of osteogenic induction, the cells were fixed with 4% paraformaldehyde for 15 min and washed with ddH_2_O. The cells were then treated with the Alizarin Red S Staining Kit for Osteogenesis (Product No.: C0148S, Beyotime). The specimens were observed under an optical microscope, and representative images were captured.

### 2.8. Transcriptomic Analysis

RNA samples were collected from six independent MC3T3-E1 cell samples (control group and 0.1 µg/mL of the Eocc treatment group, *n* = 3). RNA extraction and RNA sequencing were performed by OBiO Technology Corp., Ltd. (Shanghai, China). Gene expression levels between the two groups were analyzed using the Bioconductor package edgeR. The criteria for identifying significantly differentially expressed genes (DEGs) were set as |log2FC| ≥ 1 and *p* < 0.05. A heatmap of the DEGs was generated using the heatmap package. Gene Ontology (GO) and the Kyoto Encyclopedia of Genes and Genomes (KEGG) enrichment analyses of the DEGs were conducted using the R packages clusterProfiler, enrichment, and ggplot2 to explore their biological functions and signaling pathways.

### 2.9. E-Screen Assay

MCF-7 cells were cultured in the EDM for one week to eliminate the interference of endogenous estrogen. Logarithmic phase cells were seeded into 96-well plates at a density of 8000 cells per well. After the cells adhered, the medium was replaced with a drug-containing medium, and the cells were cultured for an additional 24 h. The effect of the drug on cell viability was assessed using the CCK-8 assay.

### 2.10. Dual Luciferase Reporter Assay

HEK-293 cells were transfected with ERα and ERβ promoter luciferase reporter vectors (Genechem, Shanghai, China) using the Lipofectamine 3000 (Product No.: L3000150, Thermofisher, Shanghai, China) transfection reagent. Transfection efficiency was normalized and assessed by co-transfection with the pRL Renilla luciferase reporter vector. After transfection, cells were treated with the Eocc (0, 0.1, 1, 10 µg/mL) for 24 h. The positive control was treated with 1µM of 17β-estradiol (E2, CAS No.: 50-28-2, Product No.: E2758, Sigma-Aldrich, Shanghai, China), and the antagonist group was treated with 1 µM of ICI182,780 (CAS No.: 129453-61-8, Product No.: ab120131, Abcam, Shanghai, China) in combination with the positive drug or the Eocc. Luciferase activity was measured using a multifunctional microplate reader, and data were processed according to the manufacturer’s instructions of the Dual Luciferase Reporter Gene Assay Kit (Product No.: KTA8010, Abbkine, Wuhan, China).

### 2.11. Real-Time qPCR

Osteogenic induction was performed as previously described. After 7 days of induction, total RNA was isolated using Trizol and reverse transcribed into complementary DNA (cDNA) according to the manufacturer’s instructions. Relative gene expression was measured using the SYBR Green Master Mix (Yeasen, Shanghai, China) and the Fluorescent quantitative PCR instrument (Bio-Rad, CFX96 Touch). The primer sequences used are listed in Supplementary Materials, Table S1.

### 2.12. Western Blot Analysis

Total protein was extracted from cells and femoral tissue using the RIPA lysis buffer containing protease and phosphatase inhibitors. Protein concentration was determined and normalized using a BCA protein assay kit (Elabscience). Protein samples were separated by SDS-PAGE and transferred to PVDF membranes. After blocking with 5% BSA at room temperature for 2 h, each blot was incubated with primary antibodies against Osteocalcin (OCN) (Wanlei bio, WLH4378, 1:1000), Runx2 (db16015, 1:1000), Osteopontin (OPN) (db15106, 1:1000) and BSP (db16122, 1:1000) sourced from Diagbio, β-actin (AF7018, 1:1000), ERα (AF6058, 1:1000), p-ERα (AF3061, 1:1000), Akt (AF6261, 1:1000), p-AKT (AF0016, 1:1000), p-PI3K (AF3241, 1:1000), cleaved-caspase-9 (AF5240, 1:1000), and cleaved-caspase-3 (AF7022, 1:1000) purchased from Affinity, PI3K (A23303, 1:1000), GSK3β (A2081, 1:1000), p-GSK3β (AP1088, 1:1000), mTOR (A25581, 1:1000), p-mTOR (AP0115, 1:1000), Src (A0324, 1:1000), p-Src (AP1027, 1:1000), p70S6K (A2190, 1:1000), and p-p70S6K (AP0564, 1:1000) purchased from Abclonal. The membranes were then incubated with secondary antibodies diluted at 1:5000 at room temperature for 1 h. Finally, chemiluminescence was detected using ECL Ultra (Glpbio), and the luminescence intensity was measured using the AL600RGB imaging system (General Electric, Boston, MA, USA). ImageJ 1.8.0 software was used to quantify the grayscale values of protein bands. Protein expression levels in each group were normalized to β-actin expression levels.

### 2.13. Flow Cytometric Analysis

Apoptotic cells were quantified using the Annexin VFITC apoptosis kit (Product No.: BB-4102, Bestbio, Guangzhou, China) [33]. MC3T3-E1 cells were seeded in 6-well plates at a density of 1 × 10^5^ cells per well. After treatment with drugs for 24 h, cells were collected, washed with PBS, and resuspended in 400 µL of the binding buffer. Next, 5 µL of Annexin V-FITC was added to the cell suspension. After incubating on ice for 15 min, 10 µL of the PI staining solution was added. The cell suspension was then incubated in the dark for an additional 5 min and analyzed by flow cytometry.

### 2.14. Histological Analysis

After micro-CT analysis, the femurs were decalcified, dehydrated, embedded, and sectioned into paraffin slices. The sections were subjected to H&E staining, Masson staining, and TUNEL staining. For immunohistochemical staining, the sections were deparaffinized and underwent antigen retrieval. Briefly, the sections were incubated overnight at 4 °C with primary antibodies against Runx2 (db16015, 1:200), OCN (WLH4378, 1:200), OPN (db15106, 1:200), BSP (db16122, 1:200), cleaved-caspase-3 (AF7022, 1:200), and cleaved-caspase-9 (AF5240, 1:200). The sections were then incubated with appropriate secondary antibodies. Finally, the sections were stained with DAB, and the bone matrix was counterstained with hematoxylin. For immunofluorescence staining, the sections were incubated overnight at 4 °C with an anti-p-ERα antibody, followed by incubation with a secondary antibody at 37 °C for 1 h. The slides were then stained with DAPI and mounted with an antifade reagent. Images for H&E, Masson, and immunohistochemistry were obtained using a slide scanner (3DHISTECH). TUNEL and immunofluorescence images were captured using a Zeiss LSM880 confocal microscope. Quantitative analysis was performed using ImageJ 1.8.0 software.

### 2.15. Statistical Analysis

Data are presented as mean ± SD. All analyses were performed on at least three samples or independent experiments. Statistical analyses were conducted using GraphPad Prism (version 8.0, GraphPad Software, San Diego, CA, USA). The *p*-values were calculated using unpaired Student’s *t*-tests for normally distributed samples and one-way analysis of variance (ANOVA) for multiple comparisons. Tukey’s post hoc test was used for group comparisons following ANOVA. A *p*-value of <0.05 was considered statistically significant.

## 3. Results

### 3.1. Eocc Enhances Bone Density and Osteogenic Activity in OVX-Induced PMOP Mice

**Figure 1:**
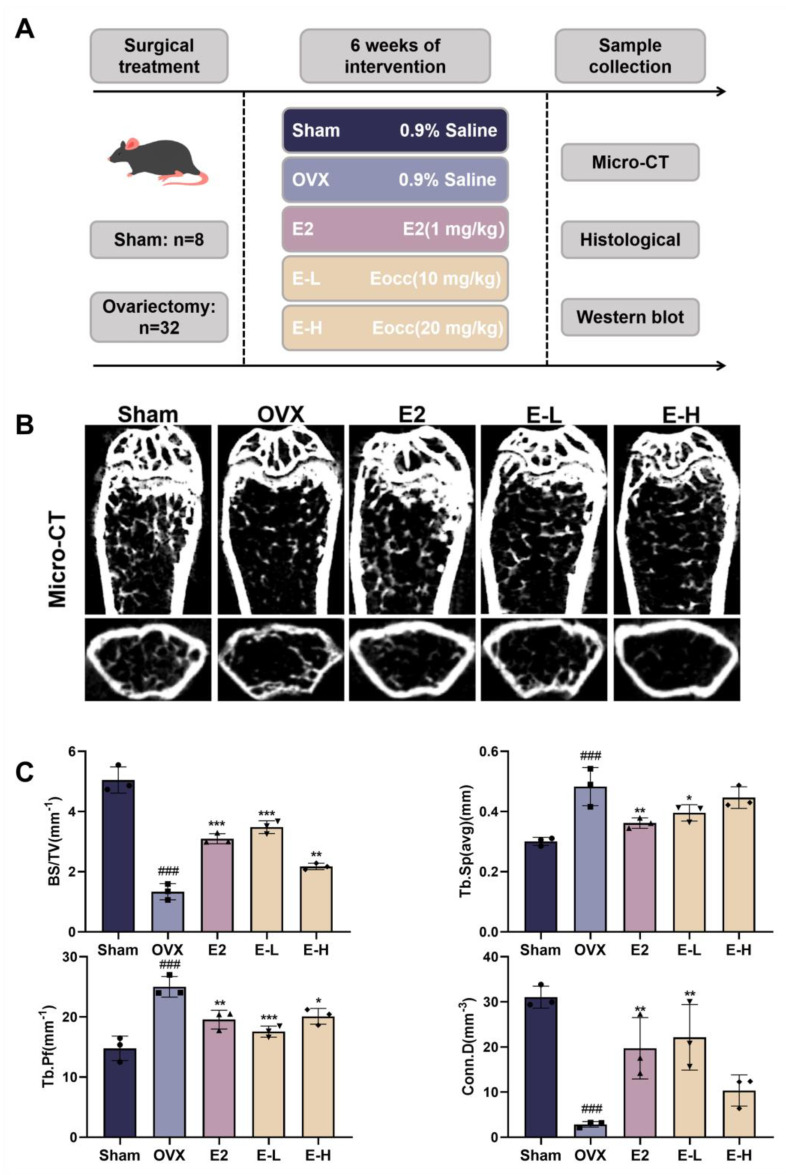
Eocc improves bone-related parameters in PMOP mice: (**A**) Schematic representation of the PMOP mouse model and treatment protocol. (**B**,**C**) Representative micro-CT images of the distal femur and bone parameter analysis for different groups. All bar graphs show mean ± SD. All bar graphs show mean ± SD. Comparisons to the OVX group are as follows: * *p* < 0.05, ** *p* < 0.01, *** *p* < 0.001; comparisons to the Sham group are as follows: ### *p* < 0.001.

**Figure 2:**
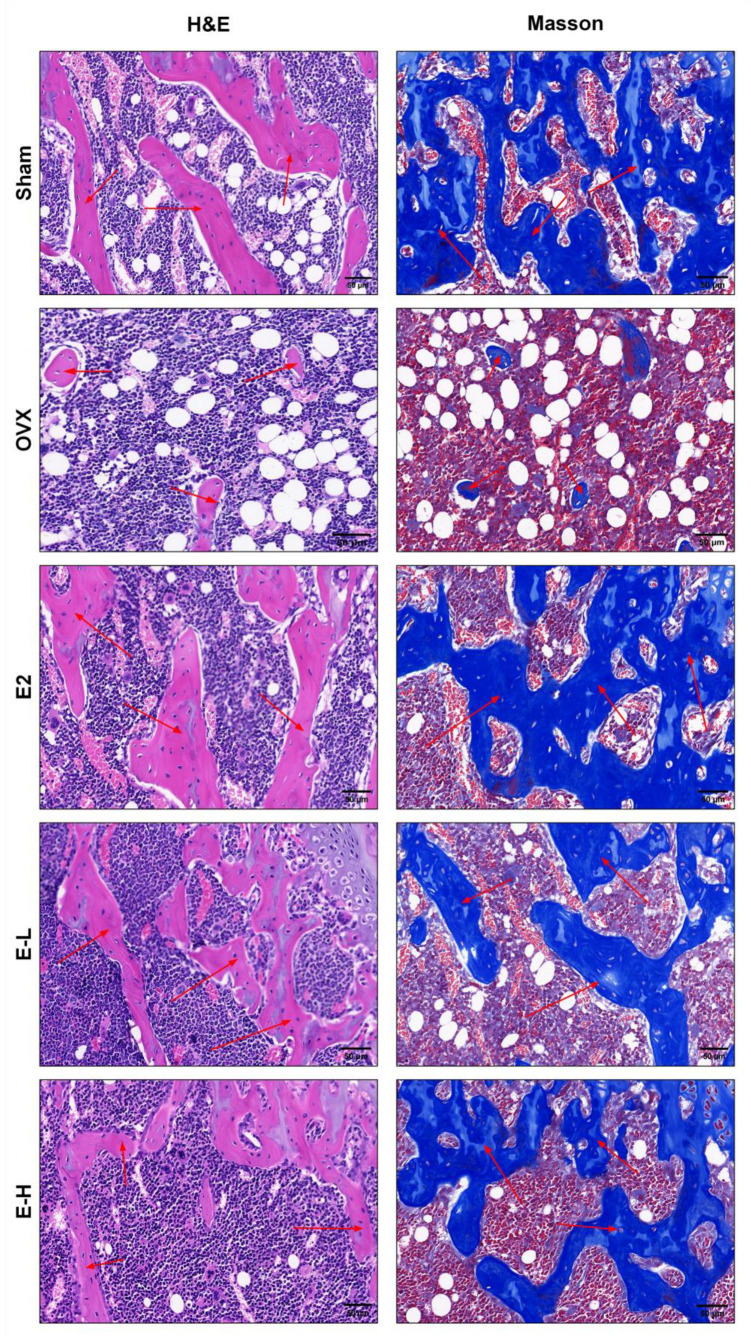
Changes in H&E staining and Masson staining at the distal femur of mice after Eocc treatment. Representative micrographs of H&E and Masson-stained sections, magnified at 40× with a scale bar of 50 µm. The red arrows in the figure indicate bone trabeculae.

**Figure 3:**
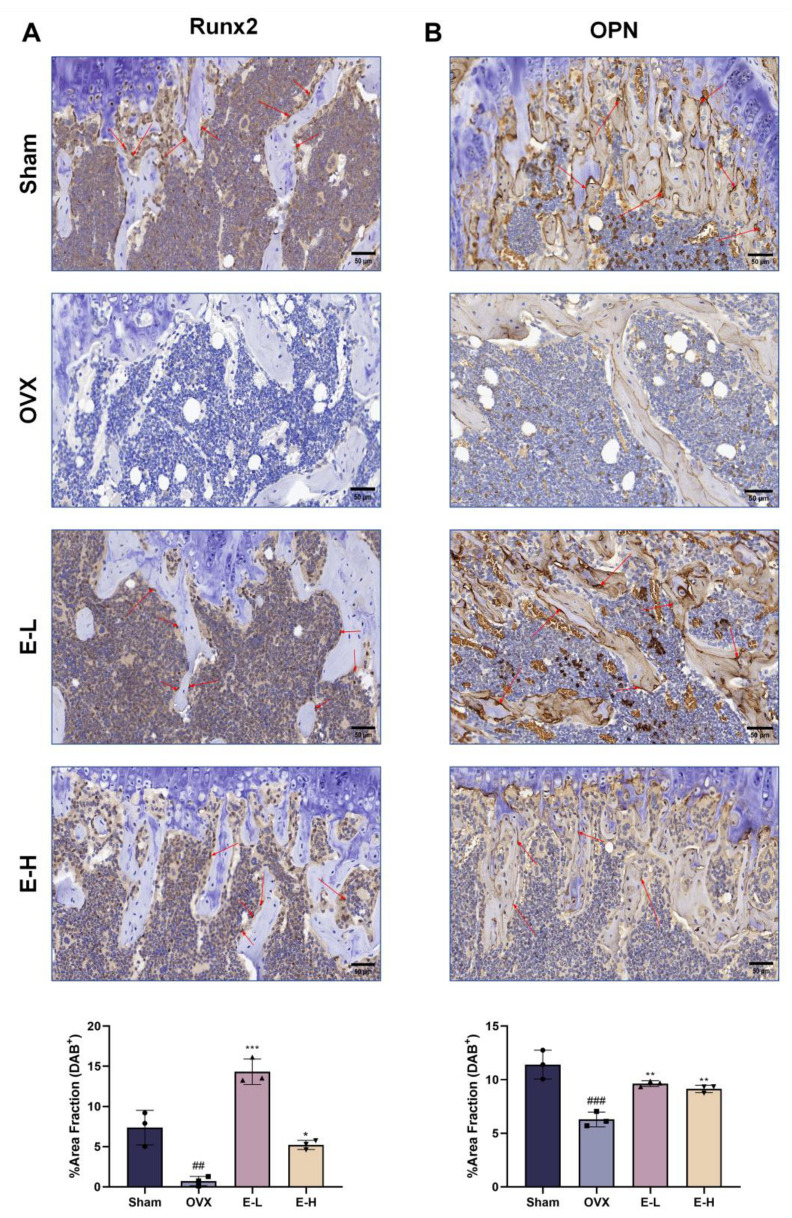

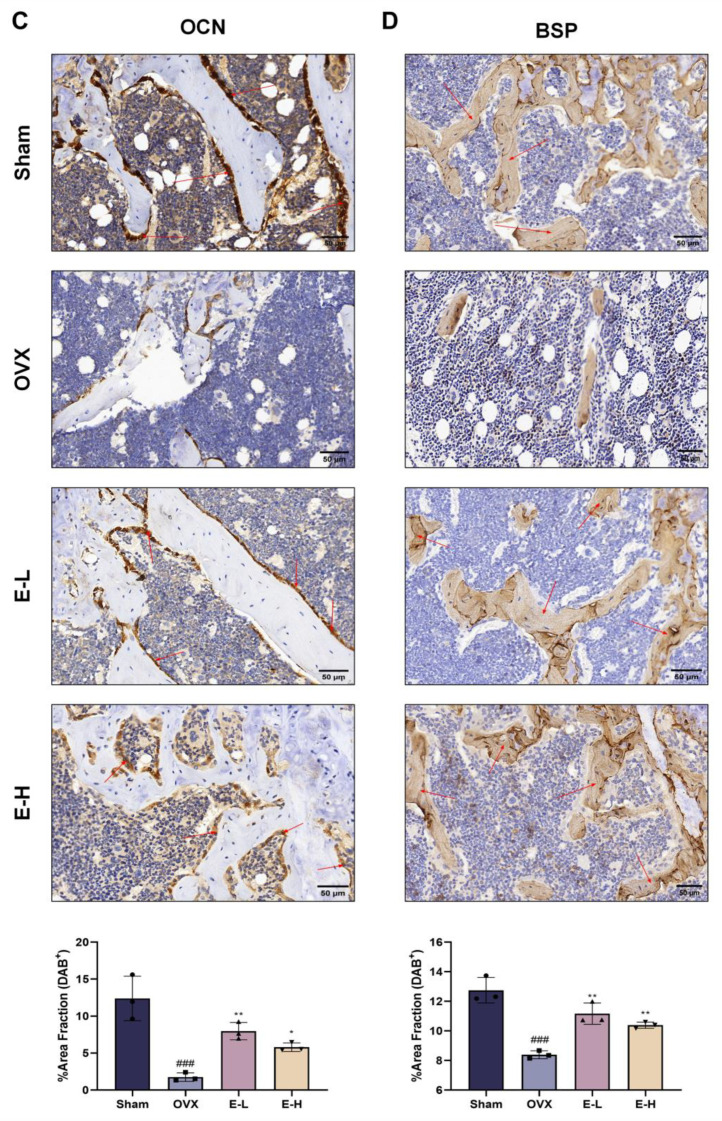
Eocc promotes the expression of osteogenic marker proteins in PMOP mice: (**A**,**B**) Immunohistochemical staining for Runx2, OPN in paraffin-embedded sections of the distal femur, with quantitative analysis of positive staining levels in each tissue region. (**C**,**D**) Immunohistochemical staining for OCN, BSP in paraffin-embedded sections of the distal femur, with quantitative analysis of positive staining levels in each tissue region. The area indicated by the red arrows in the image represents the DAB-positive region. Magnified at 40× with a scale bar of 50 µm. All bar graphs show mean ± SD. Comparisons to the OVX group are as follows: * *p* < 0.05, ** *p* < 0.01, *** *p* < 0.001; comparisons to the sham group are as follows: ## *p* < 0.01, ### *p* < 0.001.

To evaluate the therapeutic effects of the Eocc on PMOP in vivo, a PMOP mouse model was established via OVX surgery (Figure 1A). After OVX, the mice were treated with the Eocc and E2 for 6 weeks. Throughout the experiment, there were no significant differences in body weight changes among the groups (Figure S2). Micro-CT was employed to assess the microstructural characteristics of bone. The results revealed a reduction in bone mass in OVX mice compared to the sham group (Figure 1B). BV/TV represents the ratio of bone tissue volume to total tissue volume, directly reflects changes in bone volume. Among the treatment groups, the Eocc administered at a dose of 10 mg/kg exhibited the most pronounced anti-osteoporotic effects. Following drug treatment, Conn.D, Tb.Sp, and Tb.N, indicators of trabecular bone health, showed significant improvements, suggesting that the Eocc enhances trabecular bone connectivity and quantity, ultimately leading to increased bone density (Figure 1C). H&E staining results also confirmed that treatment with the Eocc restored the trabecular structure in the OVX group. Masson staining demonstrated that the trabecular bone in sham mice exhibited normal morphology characterized by a dense and well-organized structure, with thick trabeculae and a prominent osteoblast presence, indicative of healthy bone tissue. Conversely, the OVX group displayed sparse and discontinuous trabecular bone, highlighting the adverse effects of ovariectomy on bone architecture (Figure 2). Immunohistochemical analysis revealed the reduced expression levels of osteogenesis-related markers, including Runx2, OPN, OCN, and BSP, in the OVX mice. Notably, treatment with the Eocc led to a marked upregulation of these markers, suggesting enhanced osteogenic activity and supporting the therapeutic efficacy of the Eocc in promoting bone formation (Figure 3).

The Eocc was found to increase bone mass in OVX-induced PMOP mice, enhance the number of trabeculae, improve trabecular connectivity, and promote the expression of osteogenic markers. These results indicate its potential to effectively stimulate bone formation in vivo.

### 3.2. Eocc Improved Osteoblast Proliferation, Differentiation, and Mineralization in MC3T3-E1 Cells

**Figure 4:**
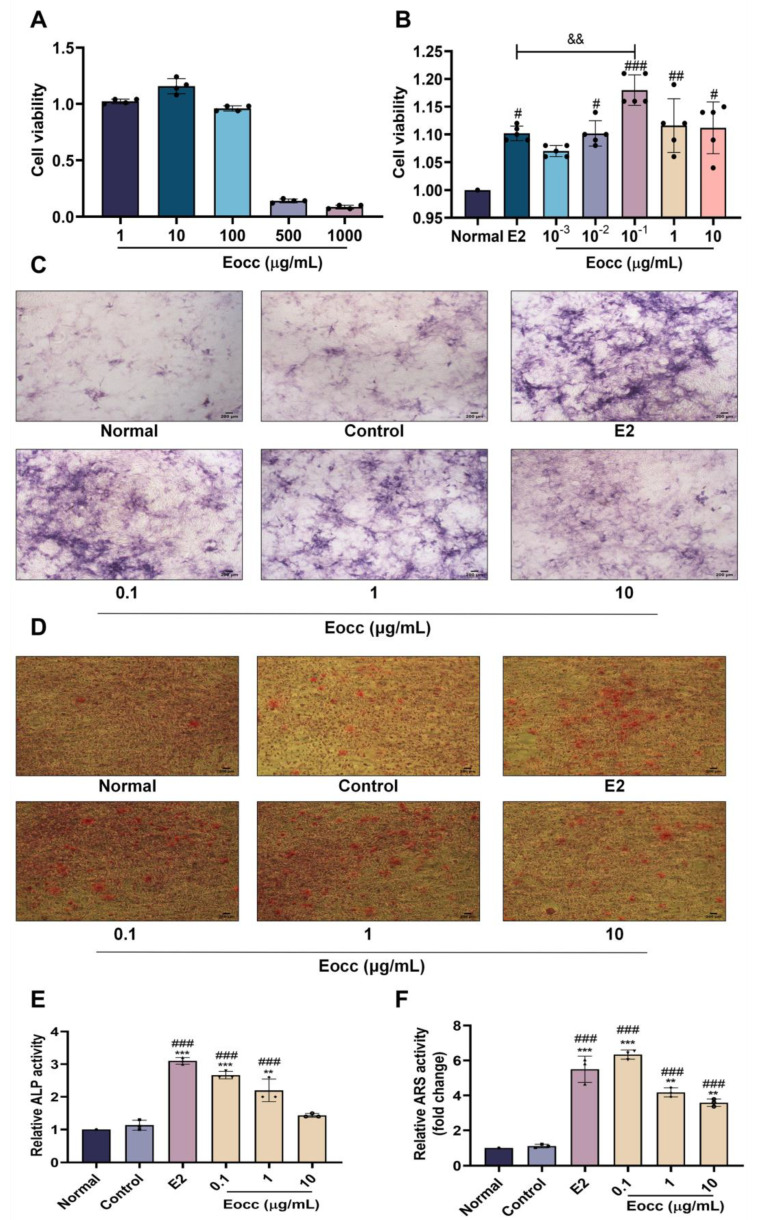
The osteogenic effects of the Eocc in vitro: (**A**) The CCK-8 assay was used to evaluate the cytotoxicity of the Eocc on MC3T3-E1 cells at concentrations ranging from 1 to 1000 μg/mL (*n* = 3). (**B**) The CCK-8 assay shows the effect of the Eocc on MC3T3-E1 cell viability at concentrations ranging from 0.001 to 10 µg/mL (*n* = 5). (**C**,**E**) Representative ALP staining images with a scale bar of 200 µm, showing that the Eocc and 17β-estradiol significantly enhance ALP activity (*n* = 3). (**D**,**F**) Representative images of osteogenic mineralized nodule staining, indicating that the Eocc and 17β-estradiol promote mineralized nodule formation (*n* = 3). Magnified at 40× with a scale bar of 200 µm. All bar graphs show mean ± SD. Comparisons to the control group are as follows: ** *p* < 0.01, *** *p* < 0.001; comparisons to the normal group are as follows: # *p* < 0.05, ## *p* < 0.01, ### *p* < 0.001; and comparisons to the E2 group are as follows: && *p* < 0.01.

The CCK-8 assay was first used to assess the cytotoxic effects of the Eocc on MC3T3-E1 cells. The results showed that the Eocc had no impact on the growth of MC3T3-E1 cells at concentrations of 1, 10, and 100 μg/mL (Figure 4A). To evaluate the effects of the Eocc on osteoblast proliferation and differentiation, MC3T3-E1 cells were treated with the Eocc at concentrations of 0.001, 0.01, 0.1, 1, and 10 µg/mL. The CCK-8 assay demonstrated that the Eocc significantly increased cell viability, with the 0.1 µg/mL concentration exhibiting the most potent proliferative effect (Figure 4B). Therefore, the concentrations of 0.1, 1, and 10 µg/mL were selected for further analysis of osteoblast differentiation. ALP activity and Alizarin red staining were employed to assess the osteogenic differentiation and mineralization potential of the Eocc in MC3T3-E1 cells. Treatment with the Eocc significantly enhanced ALP activity, with the 0.1 µg/mL concentration showing a stronger effect than that of E2. At higher concentrations (1 and 10 µg/mL), the ALP activity promoted by the Eocc was lower compared to the effect at 0.1 µg/mL and was no longer comparable to that of E2 (Figure 4C,E). Similarly, Alizarin red staining revealed that Eocc treatment significantly promoted mineralization at all tested concentrations. However, only at 0.1 µg/mL did the Eocc exhibit an effect comparable to that of E2, while the mineralization effect at higher concentrations (1 and 10 µg/mL) was reduced (Figure 4D,F).

**Figure 5:**
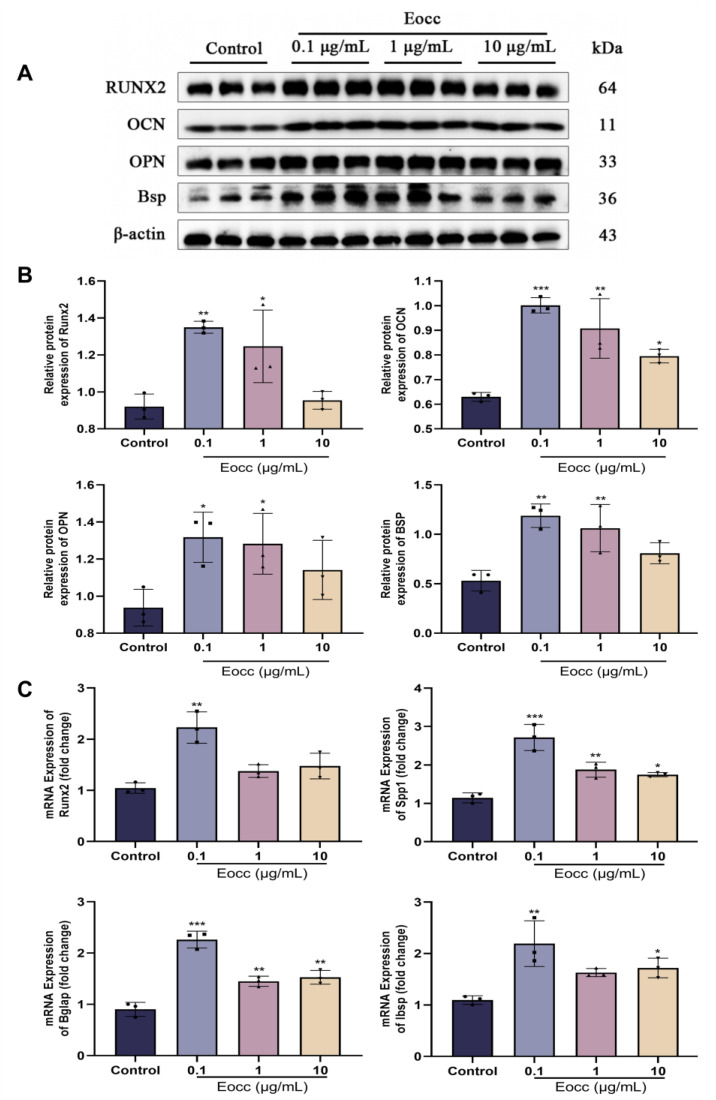
The osteogenic effects of the Eocc in vitro: (**A**,**B**) Western blot analysis of Runx2, OCN, OPN, and BSP proteins at different Eocc concentrations (0, 0.1, 1, 10 µg/mL), with quantitative analysis (*n* = 3). (**C**) PCR analysis of the osteogenic marker gene expression at various Eocc concentrations (0, 0.1, 1, 10 µg/mL), with quantitative analysis (*n* = 3). All bar graphs show mean ± SD. Comparisons to the control group are as follows: * *p* < 0.05, ** *p* < 0.01, *** *p* < 0.001.

Western blotting was then performed to assess the protein expression levels of key osteogenic markers, including Runx2, OPN, OCN, and BSP. The results indicated that the Eocc significantly upregulated the expression of these proteins in MC3T3-E1 cells, further confirming its osteogenic potential (Figure 5A,B). Subsequently, the qRT-PCR was used to evaluate the expression of osteogenic genes, including Runx2, SPP1, Bglap, and Ibsp. The Eocc significantly increased the mRNA expression levels of these genes, supporting the findings from Western blot analysis (Figure 5C).

Taken together, these results indicate that the Eocc effectively induces osteogenic differentiation and mineralization in MC3T3-E1 cells, with 0.1 µg/mL exhibiting the most notable activity.

### 3.3. Eocc Promotes ERα Activation and Mediates Osteogenic Effects Through an ERα-Dependent Pathway

**Figure 6:**
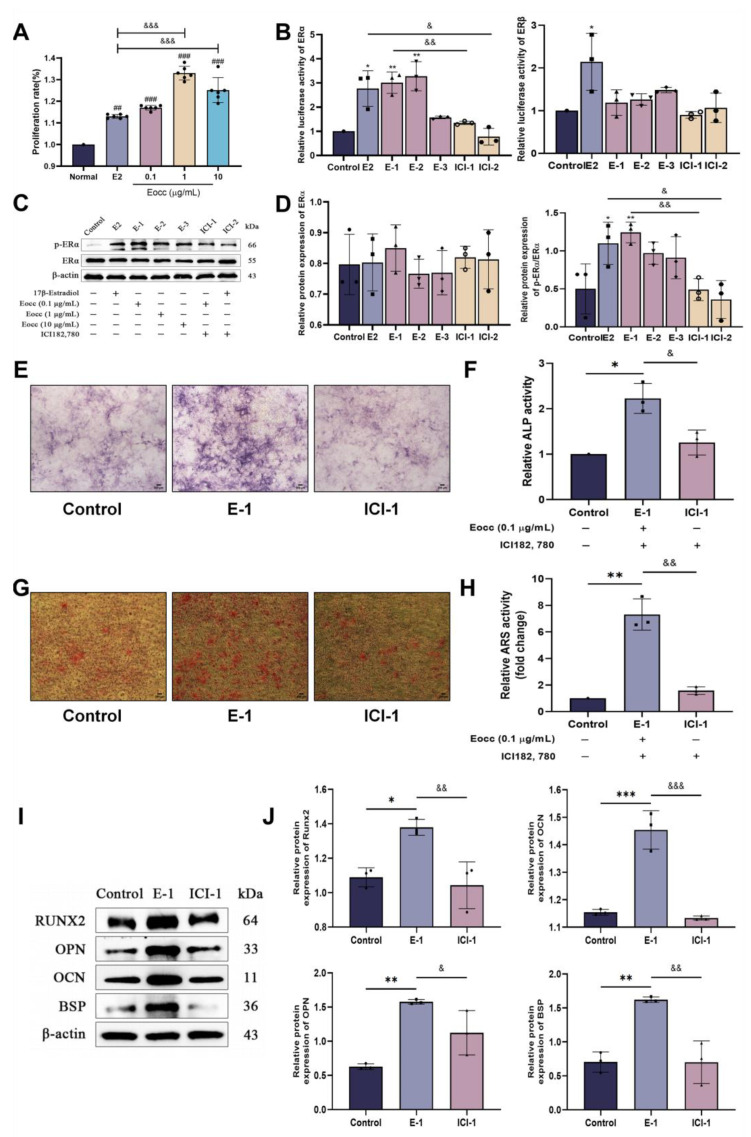
The Eocc activates ERα to promote osteogenic activity: (**A**) Cell viability of MCF-7 cells treated with different concentrations of the Eocc (0, 0.1, 1, 10 µg/mL) for 24 h, assessed by the CCK-8 assay. Treatment with 1 µM of E2 was used as a positive control. (**B**) The dual luciferase reporter assay measuring the activation of ERα and ERβ. Treatment groups included different concentrations of the Eocc (0, 0.1, 1, 10 µg/mL), positive control E2 (1 µM), co-treatment with E2 and ICI 182,780 (1 µM), and co-treatment with 0.1 µg/mL of the Eocc and ICI 182,780 (1 µM). (**C**,**D**) Western blot analysis and quantification of ERα and p-ERα expression levels. (**E**,**F**) Representative images of ALP staining (*n* = 3). Magnified at 40× with a scale bar of 200 µm. (**G**,**H**) Representative images of osteogenic mineralization nodule staining (*n* = 3). Magnified at 40× with a scale bar of 200 µm. (**I**,**J**) Western blot analysis and the quantification of osteogenic marker proteins in MC3T3-E1 cells treated with the Eocc, with or without ERα inhibition. All bar graphs represent mean ± SD. Comparisons versus the control group are as follows: * *p* < 0.05, ** *p* < 0.01, *** *p* < 0.001; versus the normal group are as follows: ## *p* < 0.01, ### *p* < 0.001; and versus the E2 or E-1 group are as follows: & *p* < 0.05, && *p* < 0.01, &&& *p* < 0.001.

Previous studies have demonstrated a close relationship between ERα and osteoblast proliferation. Therefore, we investigated whether the Eocc could promote the activation of ERα. We conducted E-screen and dual luciferase reporter assays. In the E-screen assay, MCF-7 cells were treated with varying concentrations of the Eocc for 24 h, leading to a significant increase in cell proliferation relative to the control group, indicating its estrogen-like activity. Interestingly, this proliferative effect was more pronounced than that observed with E2 (Figure 6A). In the dual luciferase reporter assay, the Eocc at 0.1 and 1 µg/mL concentrations significantly activated ERα expression, an effect that was abolished by co-treatment with the selective estrogen receptor degrader (SERD) ICI 182,780 (*p* < 0.05). However, no significant activation of ERβ was observed at any of the tested concentrations of the Eocc (Figure 6B). Western blot analysis further confirmed the activation of ERα by the Eocc (0.1 µg/mL) in MC3T3-E1 cells. Eocc treatment resulted in a slightly higher level of p-ERα compared to the E2-treated group (Figure 6C,D). These results provide robust evidence that the Eocc specifically activates ERα, but not ERβ.

Subsequently, we re-evaluated the osteogenic effects of the Eocc in the presence of ICI 182,780 to determine whether its bone-promoting activity was mediated through ERα. Specifically, we assessed ALP activity, mineralized nodule formation, and the expression of key osteogenic markers, including Runx2, OPN, OCN, and BSP. Compared to the control group, the Eocc (0.1 µg/mL) significantly increased ALP activity, stimulated mineralized nodule formation, and upregulated the expression of osteogenesis-related proteins (*p* < 0.05). However, these osteogenic effects were notably suppressed following co-treatment with ICI 182,780 (*p* < 0.05) (Figure 6E–J).

In conclusion, these findings suggest that Eocc’s osteogenic activity is primarily mediated through ERα activation, promoting bone formation via an ERα-dependent pathway.

### 3.4. Transcriptomics Reveal the PI3K/AKT Pathway as a Key Mechanism of the Eocc in Osteogenesis

**Figure 7:**
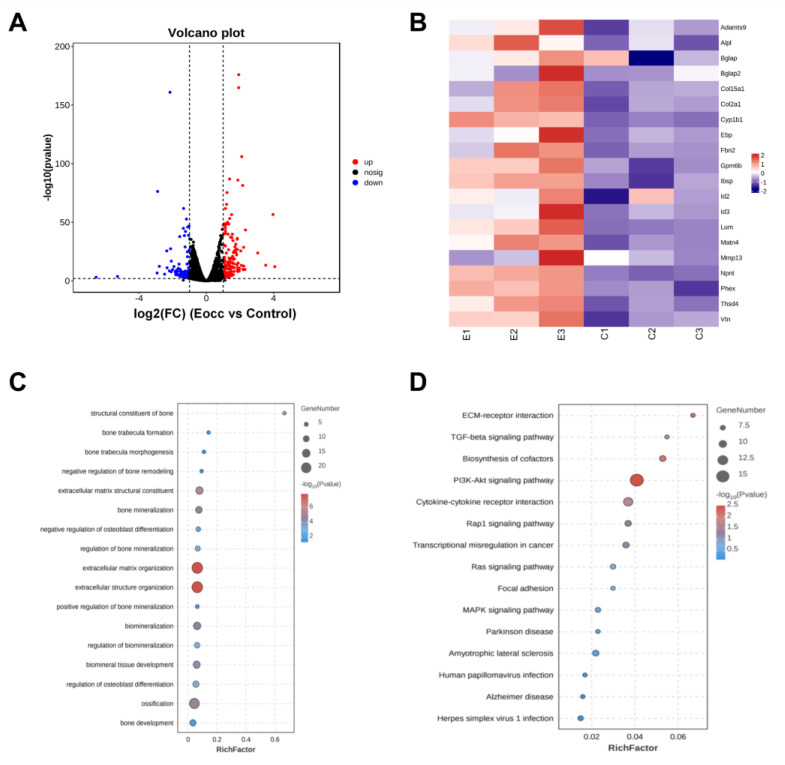
PI3K/AKT signaling may represent potential targets of Eocc in PMOP: (**A**) Transcriptomic analysis of Eocc-treated MC3T3-E1 cells. Volcano plot comparing the Eocc and control groups: blue dots represent downregulated genes, red dots represent upregulated genes, and gray dots indicate genes with no significant change in expression compared to the control group. (**B**) Heatmap of differentially expressed genes (DEGs): green indicates downregulated genes, and red indicates upregulated genes. (**C**) GO enrichment analysis of upregulated genes. (**D**) KEGG pathway enrichment analysis of upregulated genes.

Next, we investigated the mechanism of the Eocc in treating PMOP through transcriptomics. RNA-Seq analysis of Eocc-treated MC3T3-E1 cells revealed 907 differentially expressed genes, with 410 upregulated and 497 downregulated (Figure 7A). The heatmap showed significant upregulation of key osteogenic genes like Bglap, Alpl, and Ibsp after Eocc treatment, supporting its role in promoting osteogenic differentiation (Figure 7B). Further GO enrichment analysis confirmed the activation of pro-osteogenic functions, including bone trabecula formation and mineralization (Figure 7C). KEGG analysis revealed significant activation of the PI3K-AKT signaling pathway (Figure 7D).

In conclusion, based on previous studies and transcriptomic results, we hypothesize that the Eocc promotes osteogenic differentiation by activating the estrogen receptor, which in turn activates the PI3K/AKT signaling pathway.

### 3.5. In Vitro, the Eocc Activates the Src/PI3K/AKT/GSK-3β and mTOR Signaling Pathways Mediated by ERα Activation

**Figure 8:**
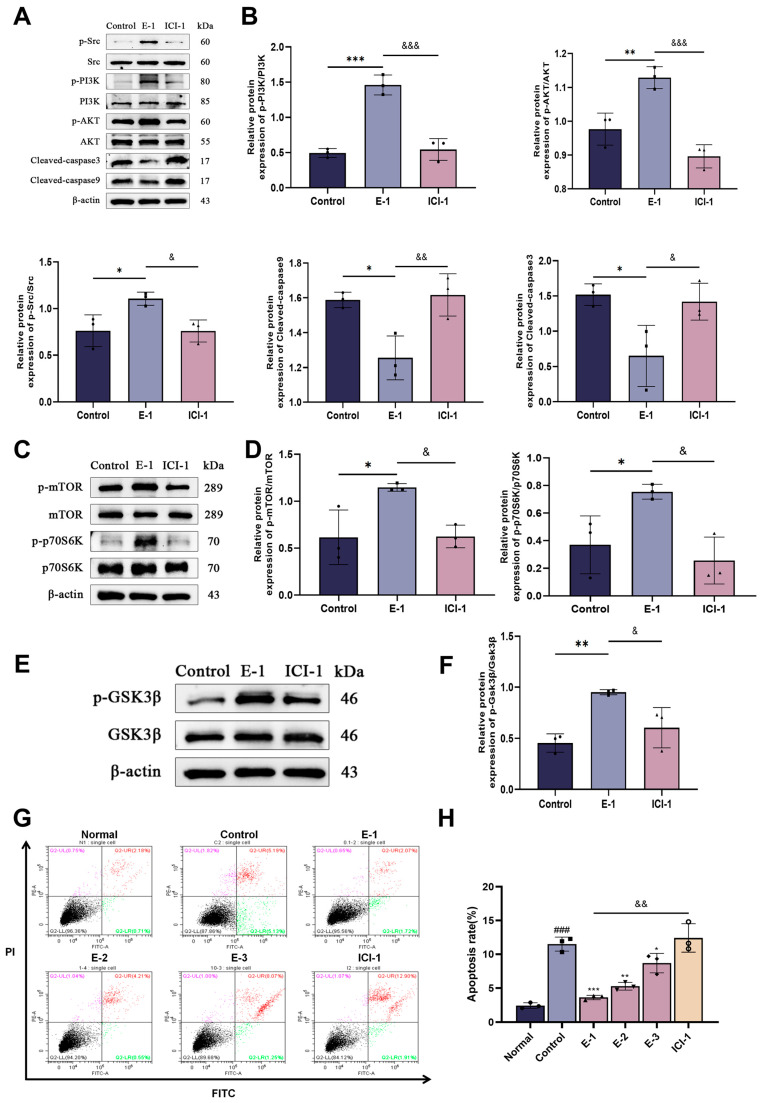
The Eocc activates the Src/PI3K/AKT and downstream signaling pathways via ERα: (**A**,**B**) Western blot analysis and the quantification of Src, p-Src, PI3K, p-PI3K, AKT, p-AKT, cleaved-caspase-3, and cleaved-caspase-9 expression levels. (**C**,**D**) Western blot analysis and quantification of mTOR, p-mTOR, p70S6K, and p-p70S6K expression levels. (**E**,**F**) Western blot analysis and quantification of GSK3β and p-GSK3β expression levels. (**G**,**H**) Representative images and quantitative analysis of cell apoptosis rates measured by flow cytometry. All bar graphs represent mean ± SD. Comparisons versus the control group: * *p* < 0.05, ** *p* < 0.01, *** *p* < 0.001; versus the normal group: ### *p* < 0.001; & *p* < 0.05, && *p* < 0.01, &&& *p* < 0.001.

Following the validation of ERα as a key target, subsequent experiments focused on the activation of the PI3K/AKT signaling pathway under this premise. To verify whether the Eocc promotes osteogenesis by activating the PI3K/AKT signaling pathway via ERα activation, we treated MC3T3-E1 cells with 0.1 µg/mL of the Eocc and 1 µM of ICI 182,780 in vitro and examined the changes in downstream signaling molecules. The results showed that 0.1 µg/mL of the Eocc significantly increased the expression of p-Src, p-PI3K, p-AKT, p-mTOR, p-p70S6K, and p-GSK3β while decreasing the expression of cleaved-caspase-3 and cleaved-caspase-9. However, treatment with 1 µM of ICI 182,780 inhibited the expression of p-Src, p-PI3K, p-AKT, p-mTOR, p-p70S6K, and p-GSK3β while increasing the expression of cleaved-caspase-3 and cleaved-caspase-9 (Figure 8A–F). These results indicate that the Eocc activates the PI3K/AKT pathway and its downstream signaling molecules and that this activation is dependent on ERα activation. Additionally, the Eocc’s promotion of the p-p70S6K expression, the inhibition of cleaved-caspase-3, and the cleaved-caspase-9 expression suggests its ability to inhibit osteoblast apoptosis and promote proliferation. Flow cytometry results further confirmed that 0.1 µg/mL of Eocc reduced the apoptosis rate of MC3T3-E1 cells, and this reduction was suppressed after ERα inhibition (Figure 8G–H).

### 3.6. In Vivo Validation of Eocc’s Role in ERα Activation and Osteoblast Survival in PMOP Mice

**Figure 9:**
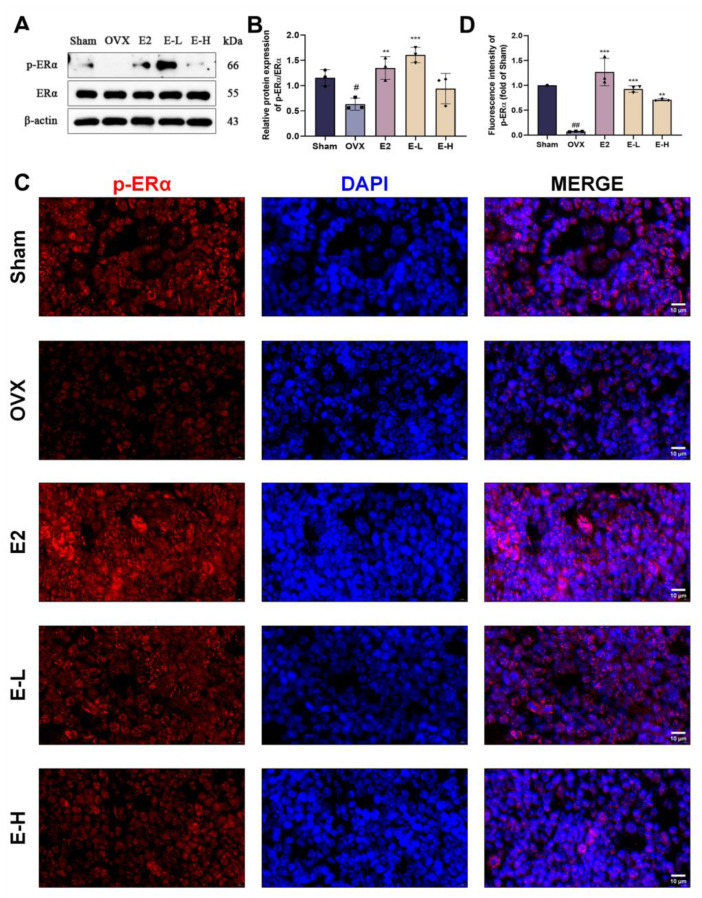
Eocc activates ERα expression in vivo: (**A**,**B**) Western blot analysis and quantification of p-ERα and ERα expression levels in mouse femur tissue. (**C**,**D**) Immunofluorescence detection of p-ERα expression in paraffin-embedded sections of the distal femur. Images are magnified at 200× with a scale bar of 10 µm. All bar graphs show mean ± SD. Comparisons to the OVX group are as follows: ** *p* < 0.01, *** *p* < 0.001; comparisons to the sham group are as follows: # *p* < 0.05, ## *p* < 0.01.

**Figure 10:**
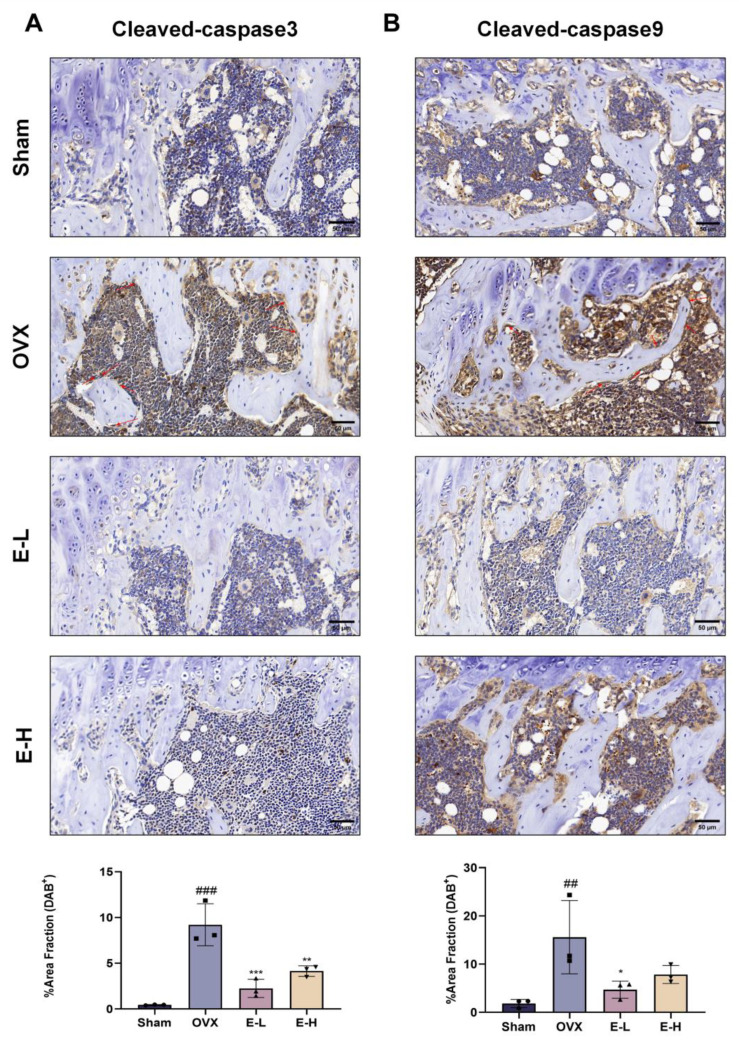
The Eocc inhibits the expression of apoptotic proteins in vivo: (**A**,**B**) Immunohistochemical staining for cleaved-caspase-3 and cleaved-caspase-9 in paraffin-embedded sections of the distal femur, with the quantitative analysis of positive staining levels in each tissue region. Images are magnified at 40× with a scale bar of 50 µm. All bar graphs show mean ± SD. Comparisons to the OVX group are as follows: * *p* < 0.05, ** *p* < 0.01, *** *p* < 0.001; and comparisons to the sham group are as follows: ## *p* < 0.01, ### *p* < 0.001.

**Figure 11:**
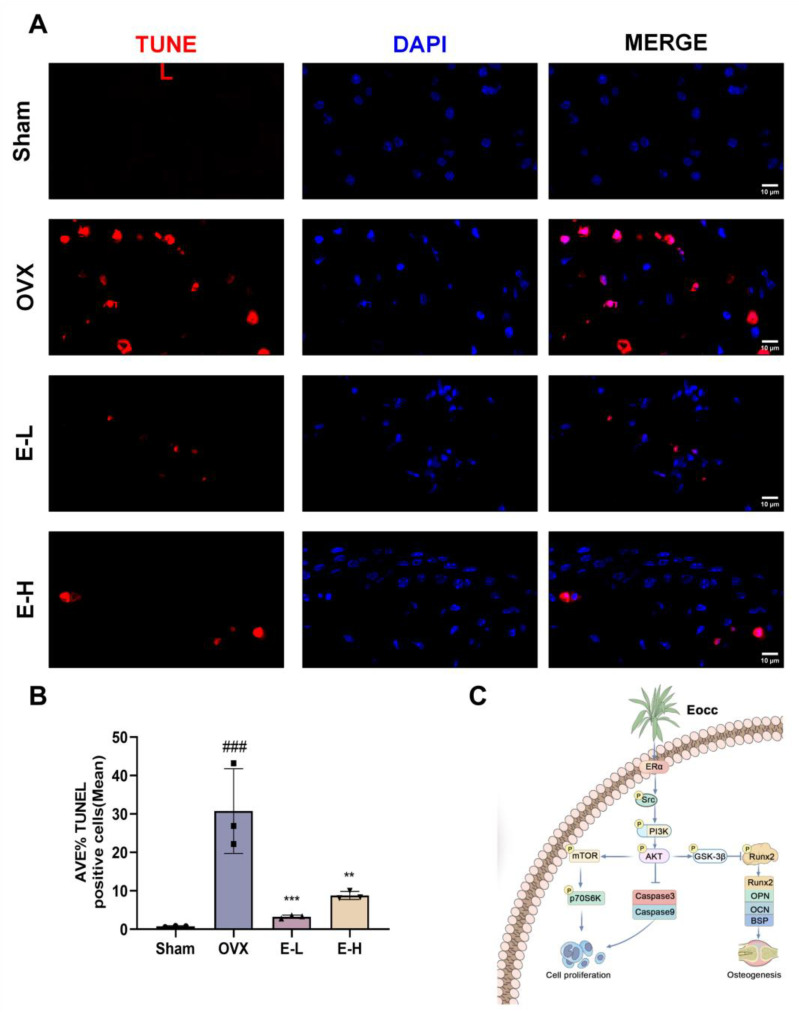
The Eocc inhibits osteoblast apoptosis in vivo: (**A**,**B**) Effect of the Eocc on TUNEL levels in mouse femur tissue. Images are magnified at 200× with a scale bar of 10 µm. (**C**) The mechanism diagram of this study. All bar graphs show mean ± SD. Comparisons to the OVX group are as follows: ** *p* < 0.01, *** *p* < 0.001; comparisons to the sham group are as follows: ### *p* < 0.001.

Moreover, we validated our experimental conclusions in vivo. Subsequently, we assessed the expression of ERα through Western blot analysis. The results showed that ERα expression levels were reduced in OVX mice, while treatment with the Eocc and E2 restored these levels, suggesting that the Eocc promotes ERα activation in PMOP mice (Figure 9A,B). Immunofluorescence for p-ERα demonstrated strong localization in both the nucleus and plasma membrane of osteoblasts in the Eocc-treated group, comparable to the E2-treated group, whereas fluorescence was weak and dispersed in the OVX group. The percentage of p-ERα-positive cells and fluorescence intensity were significantly elevated in the Eocc-treated group (*p* < 0.01), indicating that the Eocc effectively activated the ERα signaling pathway (Figure 9C,D). Additionally, the Eocc inhibited the expression of cleaved-caspase-3 and cleaved-caspase-9 in vivo (Figure 10). In TUNEL staining, a large number of TUNEL-positive cells were observed in the OVX group, primarily localized in the trabecular bone region, indicating a high level of apoptosis. In contrast, the Eocc-treated group exhibited a significant reduction in the number of TUNEL-positive cells (*p* < 0.05), with only a few weakly stained cells present, suggesting that the Eocc inhibited apoptosis in vivo (Figure 11A,B).

In summary, Eocc treatment activates ERα in PMOP mice, consistent with in vitro results. This activation leads to a significant decrease in cleaved-caspase-3 and cleaved-caspase-9 levels, inhibiting the apoptosis of osteoblasts caused by ovariectomy (OVX). Additionally, the levels of osteogenic-related proteins, such as Runx2, OPN, and OCN, also increase, as shown in Figure 3. These results suggest that the Eocc enhances bone formation by promoting osteoblast survival and differentiation through ERα activation and reduced apoptosis-related protein levels.

### 3.7. Characterization of the Chemical Constituents of the Eocc

Finally, using HPLC analysis, 22 major compounds in the Eocc were identified, with known monomeric compounds from *Curculigo capitulata* as standards. As shown in Figure S1, the chromatogram of the Eocc is presented, along with the structural formulas of the 22 identified compounds, specifically, the following: acetovanillone (C_9_H_10_O_3_, 166.06), 3,4-dihydroxybenzoic acid (C_7_H_6_O_4_, 154.02), gentisyl alcohol (C_7_H_8_O_3_, 140.04), 2,6-dimethoxy-p-benzoquinone (C_8_H_8_O_4_, 168.04), vanillic acid (C_8_H_8_O_4_, 168.04), syringic acid (C_7_H_6_O_4_, 154.02), p-coumaric acid (C_9_H_8_O_3_, 164.04), 4-hydroxybenzaldehyde (C_7_H_6_O_2_, 122.03), vanillin (C_8_H_8_O_3_, 152.04), 4-hydroxyacetophenone (C_8_H_8_O_2_, 136.05), 2,6-Dimethoxy-benzoic acid (C_9_H_10_O_4_, 182.05), coniferyl aldehyde (C_10_H_10_O_3_, 178.06), ethyl shikimate (C_9_H_14_O_5_, 202.08), sinensigenin C (C_17_H_16_O_6_, 316.09), erythro-guaiacylglycerol 8′-vanillic acid ether (C_18_H_20_O_8_, 364.11), crassifogenin B (C_17_H_12_O_6_, 312.06), curculigoside I (C_22_H_26_O_11_, 466.14), 3,4-dihydroxyphenylethyl alcohol (C_8_H_10_O_3_, 154.06), threo-5-hydroxy-3,7-dimethoxyphenylpropane-8,9-diol (C_10_H_14_O_8_, 214.08), threo-guaiacylglycerol 8′-vanillic acid ether (C_18_H_20_O_8_, 364.11), (1R,2R)-crassifogenin D (C_18_H_20_O_6_, 332.12), 4-ketopinoresinol (C_20_H_20_O_7_, 372.12). The content and retention time of the identified compounds, along with other detailed information, are presented in Supplementary Materials, Table S2. Chromatograms for all 22 compounds can be found in Supplementary Materials, Standard Spectrum.

## 4. Discussion

Postmenopausal osteoporosis is a progressive, insidious chronic metabolic bone disease primarily affecting postmenopausal women. It is characterized by the disruption of bone microarchitecture, the thinning of trabeculae, increased bone fragility, and the heightened risk of fractures [34,35]. The significant decline in estrogen levels is the primary factor driving bone loss. During postmenopausal osteoporosis, altered estrogen receptor (ER) signaling and reduced estradiol production disrupt bone homeostasis, leading to a decrease in osteoblasts and increased osteoclast activity [36]. Although current MHT, involving the use of estrogen alone or in combination with progestins, can effectively counteract postmenopausal bone loss and reduce fracture risk, positively impacting bone health, the use of MHT is associated with potential risks and side effects, including increased risks of cardiovascular events, thromboembolic diseases, and breast cancer [37]. Therefore, the search for novel drugs that can efficiently, rapidly, and safely treat estrogen deficiency and combat osteoporosis is of great importance. In this study, we demonstrated for the first time that the total extract from *Curculigo capitulata* can protect mice from ovariectomy-induced osteoporosis by promoting osteoblast proliferation and differentiation. These findings provide novel insights into the potential therapeutic effects of *Curculigo capitulata* in the treatment of PMOP.

Currently, several plants from the genus *Curculigo* are shown to exhibit anti-osteoporosis activity. Classic phenolic compounds in *Curculigo orchioides*, such as curculigoside and curculigine, have demonstrated inhibitory effects on bone resorption pit area, osteoclast formation, and TRAP activity [38]. Curculigoside protects against iron-induced bone loss by alleviating Akt-FOXO1-dependent oxidative damage in mice and MC3T3-E1 osteoblasts [19]. Orcinol glucoside promotes the differentiation of MSCs into osteoblasts and inhibits adipogenesis through the Wnt/β-catenin signaling pathway [21]. However, research on the role of *Curculigo capitulata* in the treatment of osteoporosis remains lacking.

In this study, we demonstrated that the extract of *Curculigo capitulata* can protect mice from OVX-induced osteoporosis by promoting osteoblast proliferation and differentiation. We observed that Eocc treatment significantly improved bone parameters in PMOP mice, specifically by increasing bone mass and trabecular bone density. Moreover, H&E and Masson staining showed that the trabeculae in the Eocc treatment group were thicker, more compact, and orderly arranged, with clearly visible osteoblasts. These results indicate that the Eocc can promote bone formation, increase bone density, and improve the skeletal microarchitecture in OVX mice.

Bone homeostasis is regulated by the balance between osteoblast-mediated bone formation and osteoclast-mediated bone resorption [39]. Osteoblast differentiation, as a key process in bone formation, is of great significance for the prevention and treatment of PMOP [4]. Runt-related transcription factor 2 (Runx2) is a fundamental transcription factor for bone development. In immature osteoblasts, Runx2 regulates the expression of bone matrix protein genes such as Spp1, Ibsp, and bone γ-carboxyglutamic acid protein (Bglap)/Bglap2, thereby inducing osteoblast maturation [40]. In our study, we developed an in vitro osteoblast differentiation model using MC3T3-E1 cells stimulated with 50 µg/mL of ascorbic acid and 5 mM of β-glycerophosphate. The Eocc significantly enhanced osteoblast differentiation and proliferation under these conditions and upregulated the expression of key osteogenic markers, including Runx2, OPN, OCN, and BSP.

Additionally, the bone-protective effects of estrogen are achieved by preventing osteoblast apoptosis through the inhibition of apoptosis-related gene expressions, thereby extending the lifespan of osteoblasts. Apoptosis is a key regulatory mechanism in osteoblast generation [41]. Therefore, the osteoprotective effects of the Eocc, which exhibits estrogen-like activity, may also be achieved through the inhibition of apoptosis. Flow cytometry analysis showed that Eocc treatment significantly reduced apoptosis during the induction process. Further analysis revealed that the Eocc decreased the expression of cleaved-caspase-3 and cleaved-caspase-9. These changes were also confirmed in OVX mice. Thus, both in vitro and in vivo, this suggests that the Eocc promotes bone formation by enhancing the expression of osteogenic markers, inhibiting osteoblast apoptosis, and increasing osteoblast proliferation and differentiation.

Estrogen is known to play a crucial role in bone metabolism by binding to ERs on osteoblasts, promoting their proliferation and differentiation [42]. The significance of estrogen receptors in maintaining bone homeostasis has been well documented [26,43]. Research further indicates that estrogen-like compounds interact with ERα on the plasma membrane, activating non-receptor tyrosine kinases like SRC. This activation leads to the engagement of various downstream signaling pathways, including the PI3K/AKT pathway [44,45,46]. To further elucidate the mechanism of Eocc treatment for PMOP, transcriptomics was conducted. Key findings indicated that the PI3K/AKT axis is a potential target, while osteogenic processes (including bone mineralization, bone remodeling, and ossification) are critical mechanisms influenced by the Eocc.

In this study, we found that Eocc treatment significantly upregulated ERα protein expression and notably activated the phosphorylation of Src, PI3K, and AKT. By inhibiting the estrogen receptor, we found that the osteogenic promotion and activation of the PI3K/AKT signaling pathway by the Eocc were mediated through ERα. As is well known, PI3K/AKT signaling pathway is crucial for regulating cell proliferation, differentiation, and apoptosis and has been linked to osteoporosis treatment using natural products [47,48,49,50]. The activation of this pathway promotes AKT phosphorylation, which downregulates apoptotic markers like caspase-3 and caspase-9 [51,52], enhances Runx2 activity via GSK3β inhibition [53,54], and supports mTOR-mediated cell proliferation [55,56].

After the Eocc promotes the activation of ERα/Src/PI3K/AKT, its downstream effects primarily diverge into two pathways. The first involves the osteoblast proliferation mediated by the downregulation of cleaved-caspase-3 and cleaved-caspase-9, as well as the phosphorylation of mTOR and p70S6K. The second is the inhibition of GSK3β‘s negative regulation on Runx2 expression, mediated by GSK3β phosphorylation. The Eocc, through ERα-mediated PI3K/AKT signaling and its complex downstream regulatory network, reveals the intricate mechanisms behind its promotion of osteoblast proliferation and differentiation. Eocc’s ability to regulate osteoblast proliferation and differentiation presents a promising alternative for the treatment of PMOP. The molecular mechanism by which the Eocc alleviates PMOP is illustrated in Figure 11C.

In conclusion, we demonstrated that the Eocc activates the ERα-mediated PI3K/AKT signaling pathway, promoting the proliferation and differentiation of osteoblasts, which in turn enhances bone formation in OVX mice and offers a therapeutic approach for PMOP. The elucidation of this molecular pathway opens new avenues for exploring the potential of *Curculigo capitulata* in the treatment of PMOP. However, further research and clinical trials are necessary to validate these findings and to ensure the safety and efficacy of *Curculigo capitulata* as a potential drug candidate for PMOP.

## 5. Conclusions

In summary, this study is the first to explore the positive effects of *Curculigo capitulata* on bone health and its molecular mechanisms. Previous research primarily focused on mixtures and monomers from related plants in *Curculigo orchioides*. In our study, we confirmed through in vivo and in vitro experiments that the Eocc can inhibit osteoblast apoptosis, promote osteoblast proliferation and differentiation, enhance bone formation, and improve the bone microstructure in OVX mice. Additionally, our research found that the Eocc promotes the activation of the PI3K/AKT signaling pathway through ERα activation, leading to positive changes in downstream signaling molecules that ultimately enhance osteoblast proliferation and differentiation. These findings provide new insights into the potential efficacy of the Eocc in treating osteoporosis.

## Figures and Tables

**Table 1:** Summary of animal treatments and surgical procedures.

Group	Surgical Procedure	Treatment	Dose (mg/kg)	Route of Administration	Duration
Sham	Sham surgery	saline	/	i.p.	6 weeks
Ovx	Ovariectomy	saline	/	i.p.	6 weeks
E2	Ovariectomy	17β-estradiol	1	i.p.	6 weeks
E-L	Ovariectomy	Eocc	10	i.p.	6 weeks
E-H	Ovariectomy	Eocc	20	i.p.	6 weeks

**Table 2:** Design and methodology of in vitro experiments.

Experiment Type	Cell	Group	Treatment	Culture Medium	Duration
E-Screen	MCF-7	Normal	/	CM *	24 h
		E2	17β-estradiol (1 μM)	EDM *	
		0.1	Eocc (0.1 μg/mL)	EDM	
		1	Eocc (1 μg/mL)	EDM	
		10	Eocc (10 μg/mL)	EDM	
Dual Luciferase Reporter Assay	HEK 293	Normal	/	CM	24 h
		Control	/	CM	
		E2	17β-estradiol (1 μM)	CM	
		E-1	Eocc (0.1 μg/mL)	CM	
		E-2	Eocc (1 μg/mL)	CM	
		E-3	Eocc (10 μg/mL)	CM	
		ICI-1	Eocc (0.1 μg/mL) and ICI 182,780 (1 μM)	CM	
		ICI-2	Eocc (0.1 μg/mL) and 17β-estradiol (1 μM)	CM	
Osteogenesis related experiments	MC3T3-E1	Normal	/	Specific Culture Medium	Cell proliferation and flow cytometry: 24 h; WB, qPCR, and ALP staining: 7 days; Alizarin Red staining: 21 days.
		Control	/	OIM *	
		E2	17β-estradiol (1 μM)	OIM	
		E-1	Eocc (0.1 μg/mL)	OIM	
		E-2	Eocc (1 μg/mL)	OIM	
		E-3	Eocc (10 μg/mL)	OIM	
		ICI-1	Eocc (0.1 μg/mL) and ICI 182,780 (1 μM)	OIM	

* CM: complete medium; EDM: estrogen-deficient medium; and OIM: osteogenic induction medium.

## Data Availability

Data are contained within this article.

## Supplementary Materials

The following are available online at https://www.mdpi.com/article/10.3390/cells13232028/s1, Figure S1: HPLC analysis of the Eocc. (A) HPLC chromatogram. (B) Chemical structures; Figure S2: Body weight change in mice; Table S1: The primer information of genes in this study; Table S2: Detailed information on the 22 components in the Eocc; and Standard Spectrum.
