# Supplementary material for: Extract of Curculigo capitulata Ameliorates Postmenopausal Osteoporosis by Promoting Osteoblast Proliferation and Differentiation
Source: Cells. 2024 Dec 8;13(23):2028. doi: 10.3390/cells13232028 (PMC11640542; doi:10.3390/cells13232028)
Supplement: Supplementary file 1 [file cells-13-02028-s001.zip › Figures.pdf]

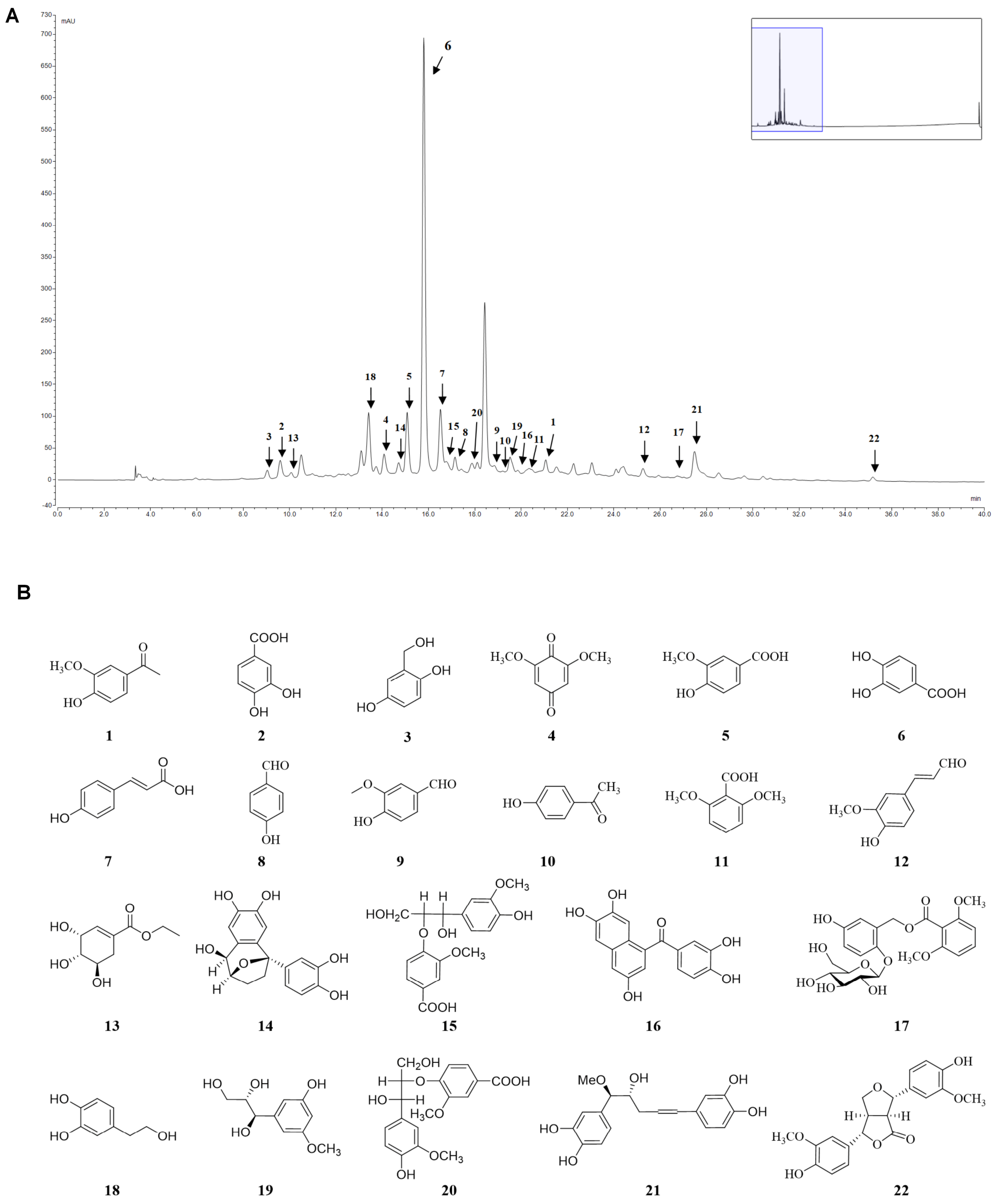

Figure S1: HPLC analysis of the Eocc. (A) HPLC chromatogram. (B) Chemical structures.

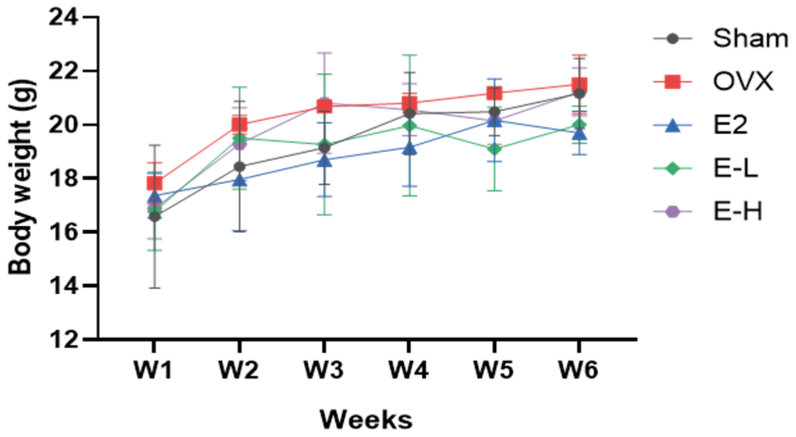

Figure S2: Body weight change in mice.
