# Supplementary material for: Extract of Curculigo capitulata Ameliorates Postmenopausal Osteoporosis by Promoting Osteoblast Proliferation and Differentiation
Source: Cells. 2024 Dec 8;13(23):2028. doi: 10.3390/cells13232028 (PMC11640542; doi:10.3390/cells13232028)

Compond **1**: acetovanillone

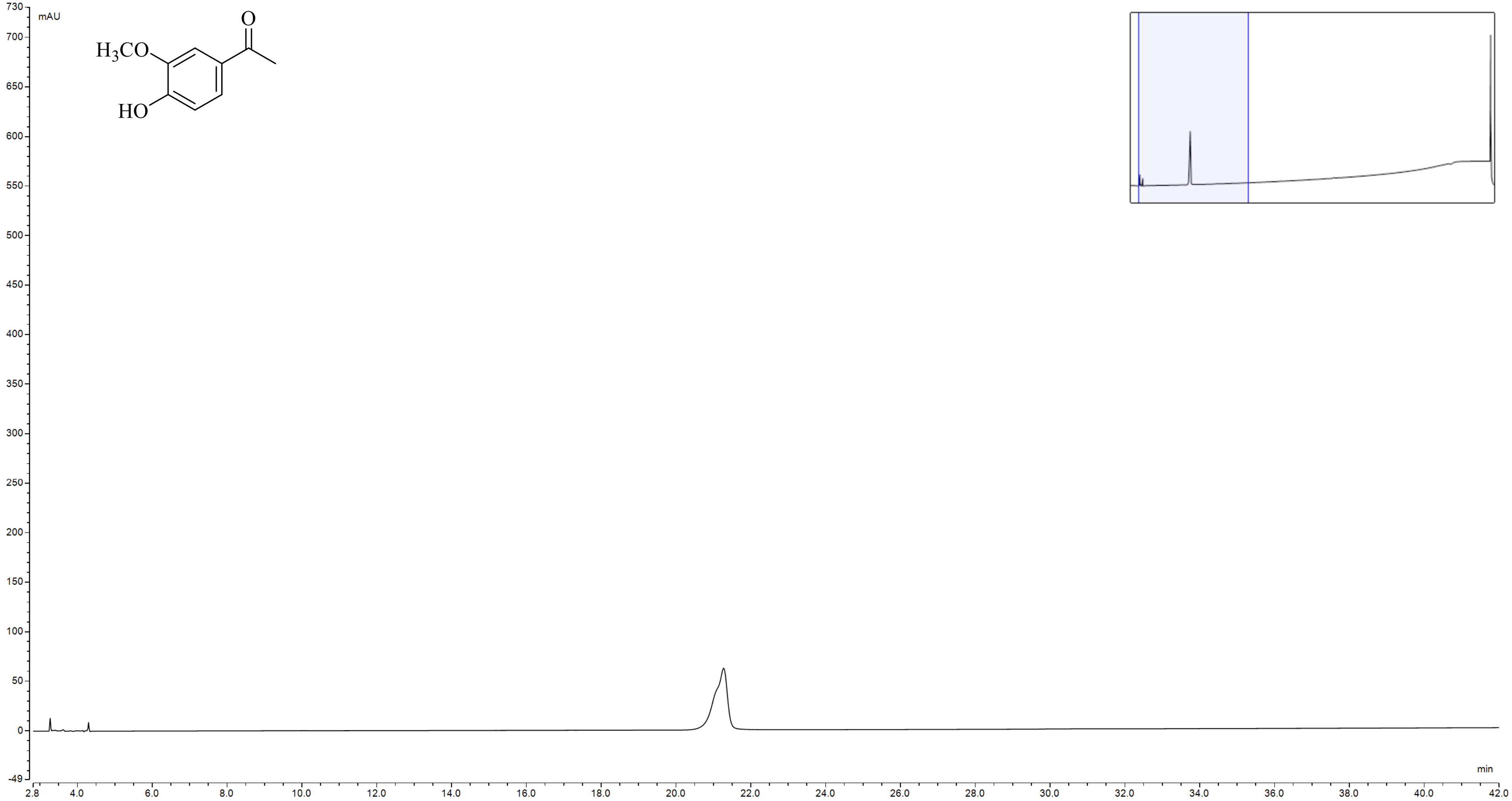

Compond **2**: 3,4-dihydroxybenzoic acid

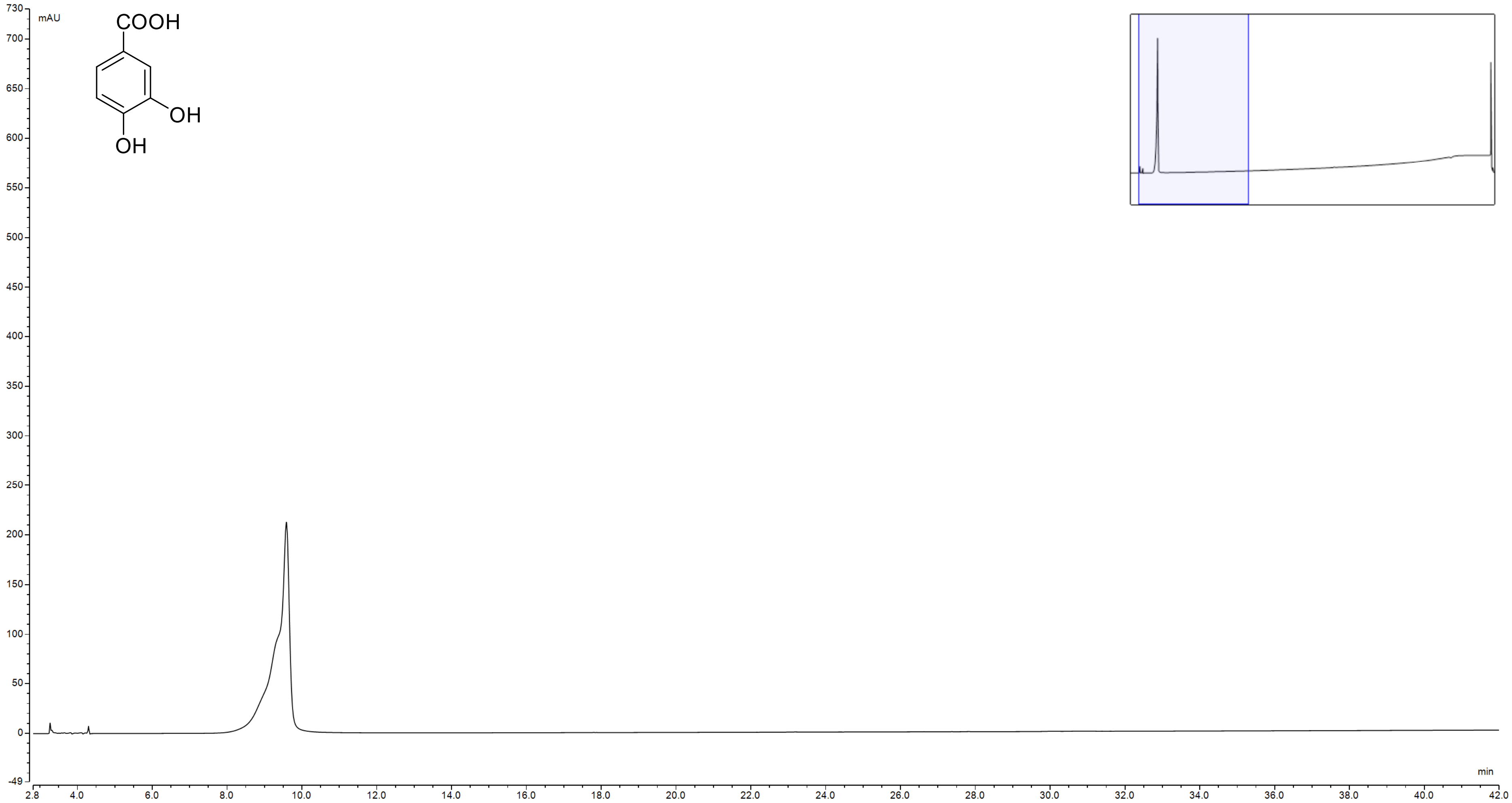

Compond **3**: gentisyl alcohol

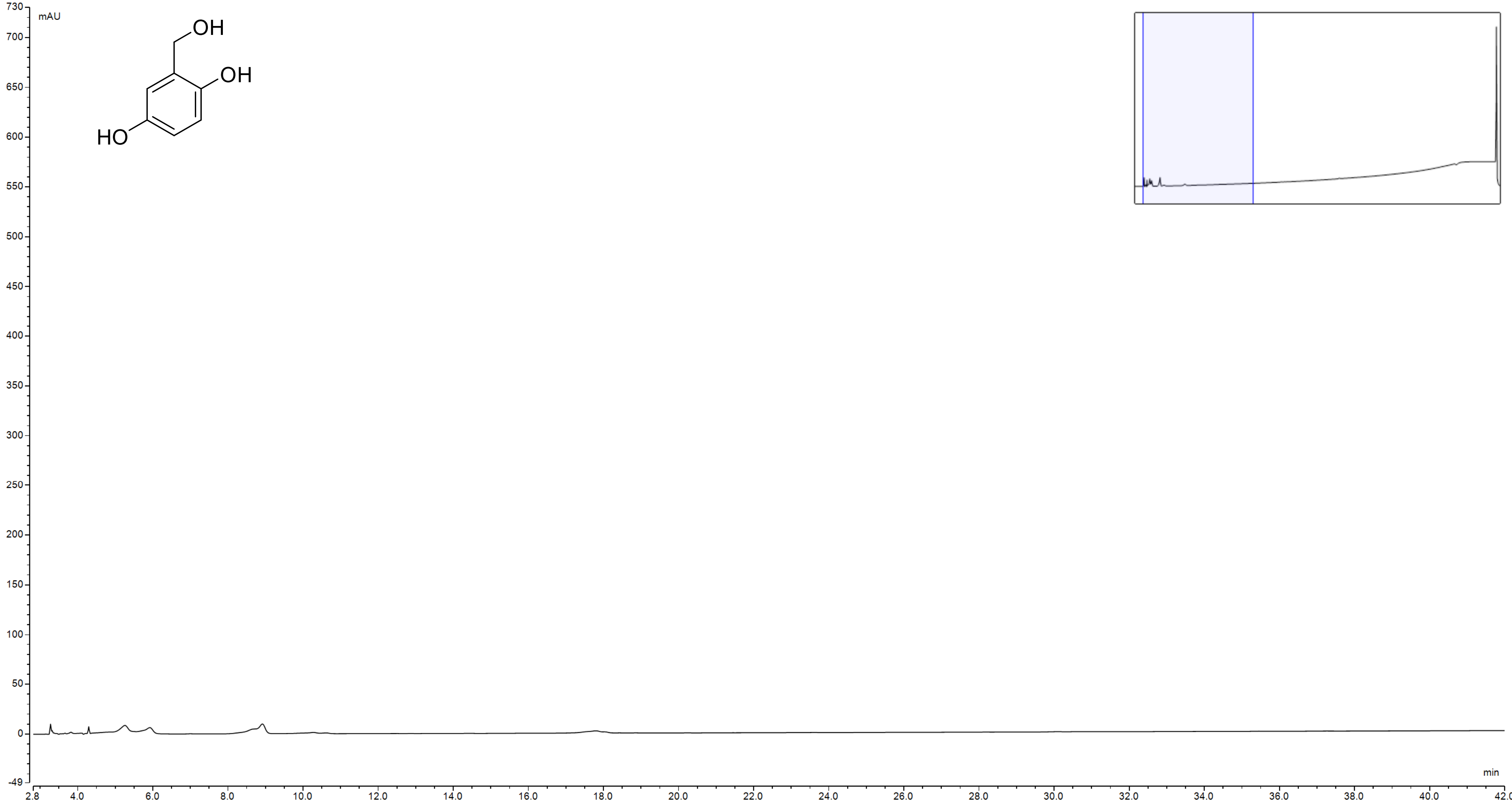

Compond **4**: 2,6-dimethoxy-p-benzoquinone

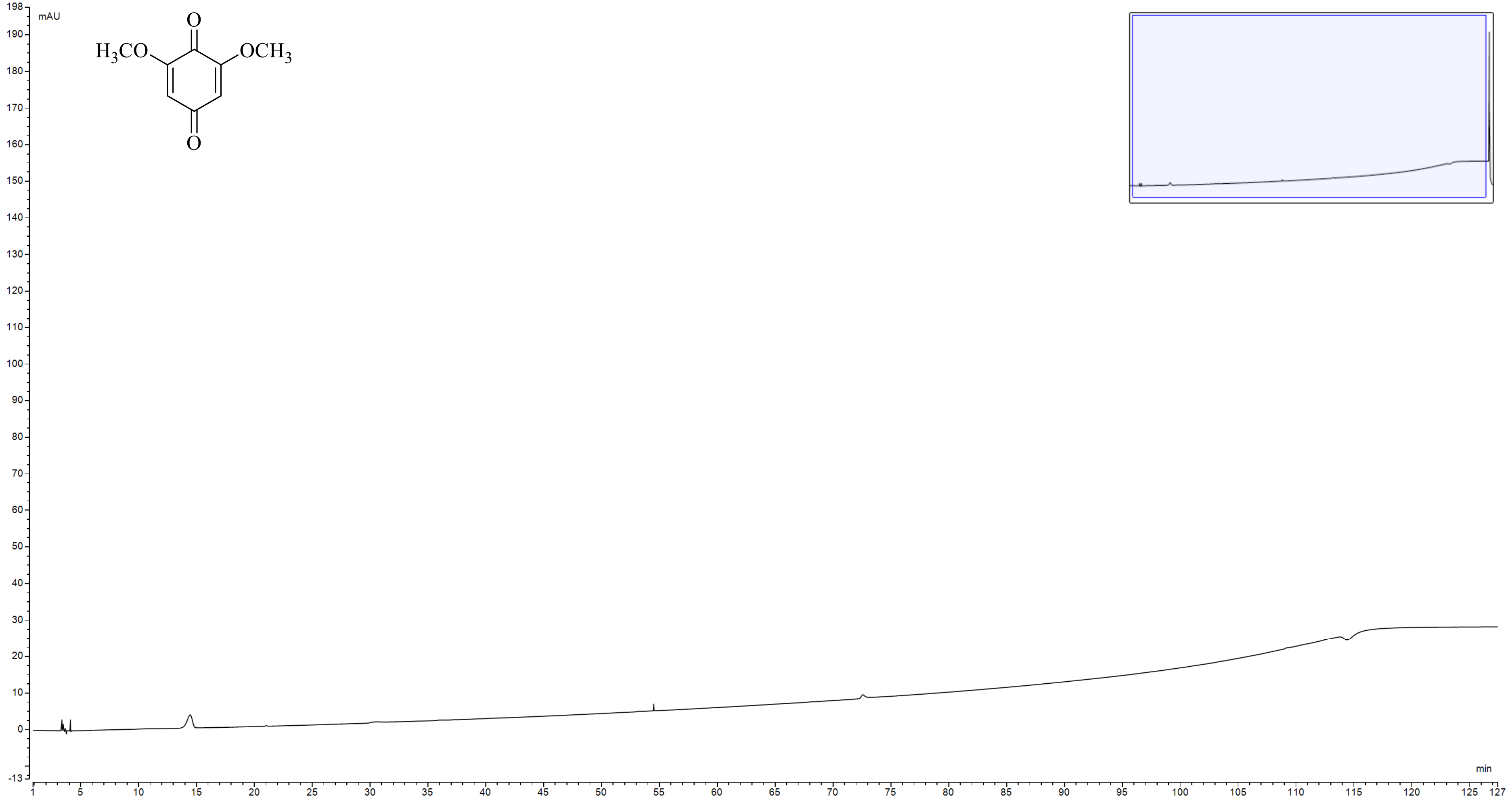

Compond **5**: vanillic acid

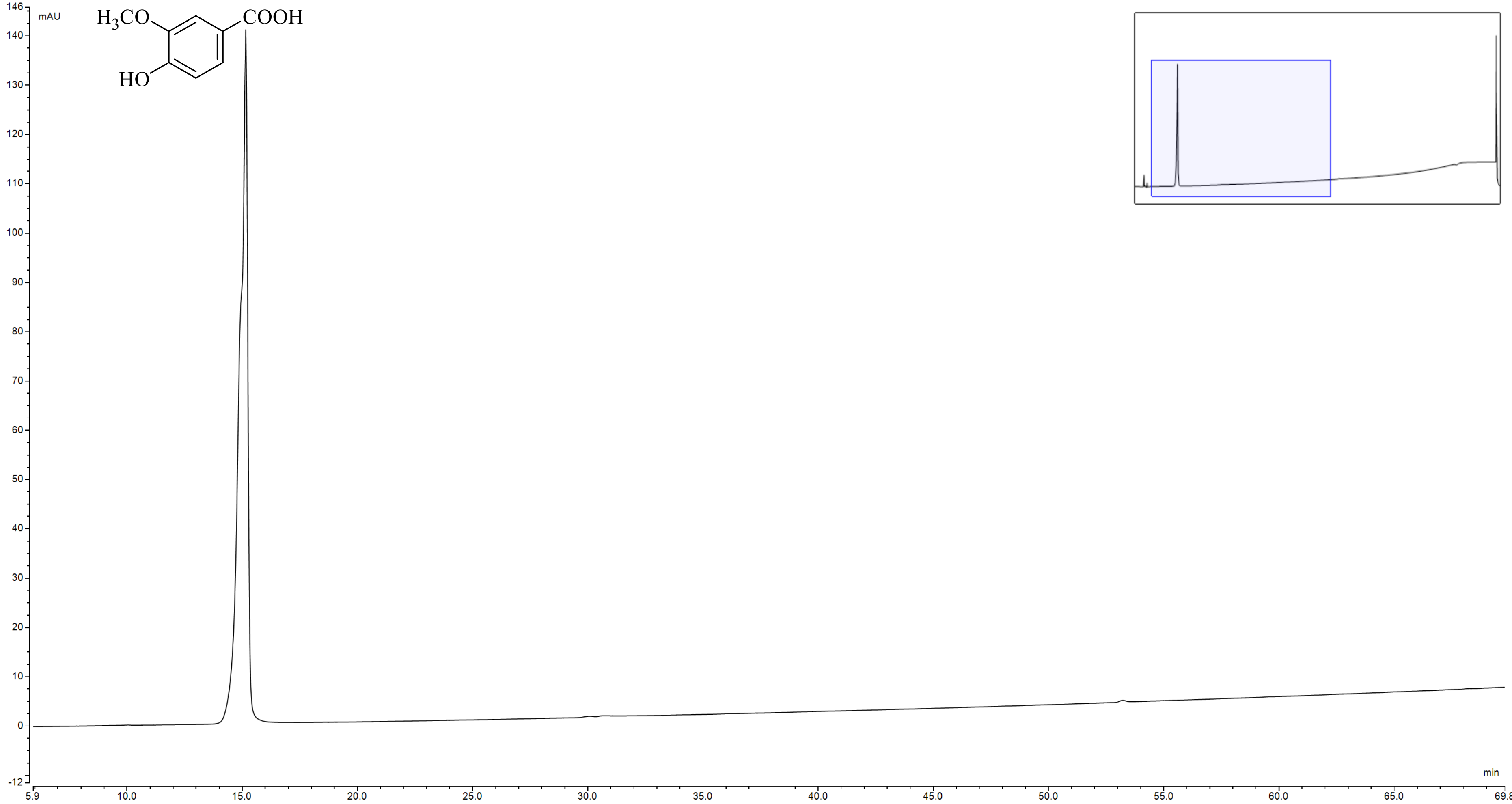

Compond **6**: syringic acid

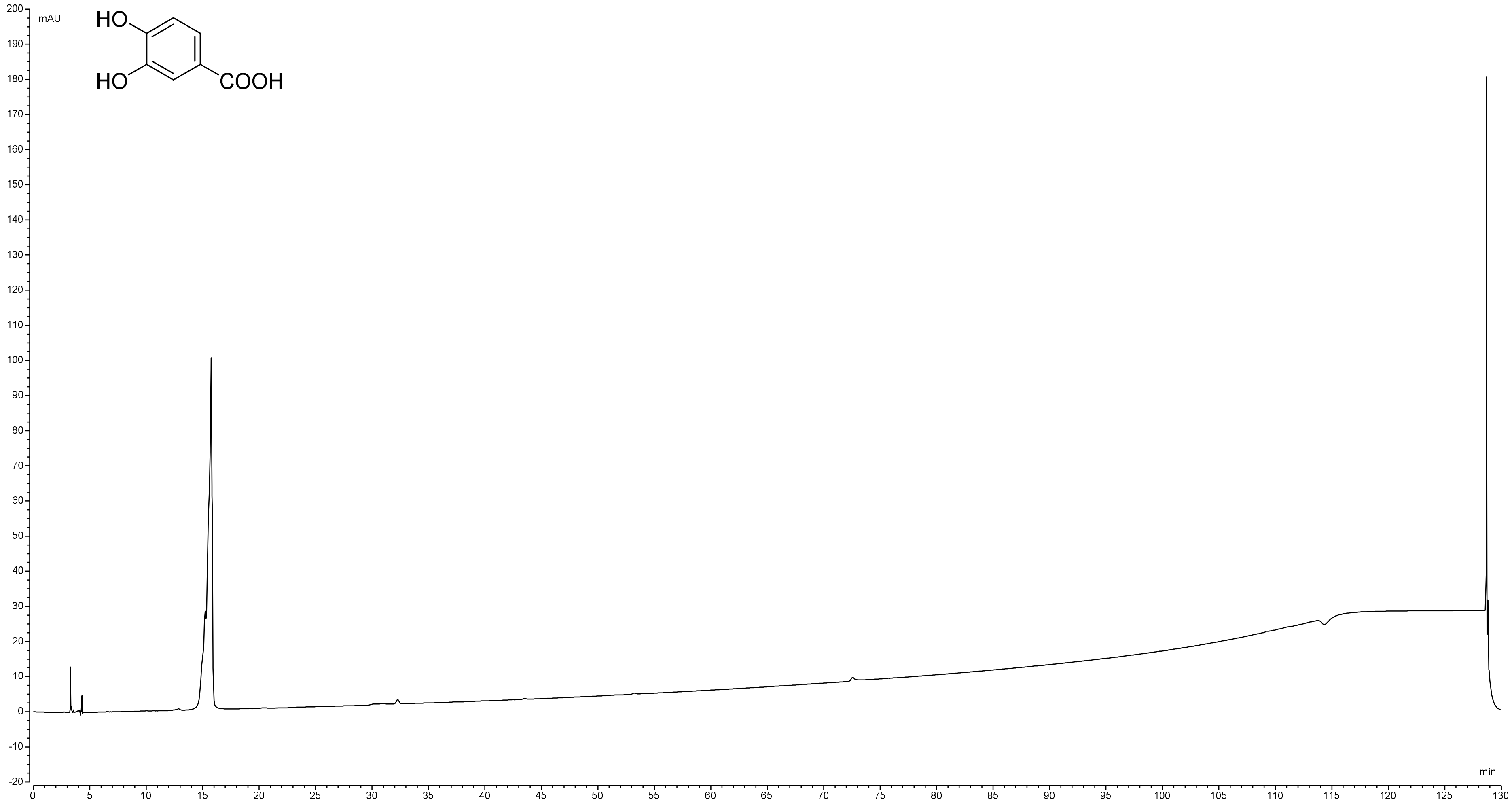

Compond **7**: p-coumaric acid

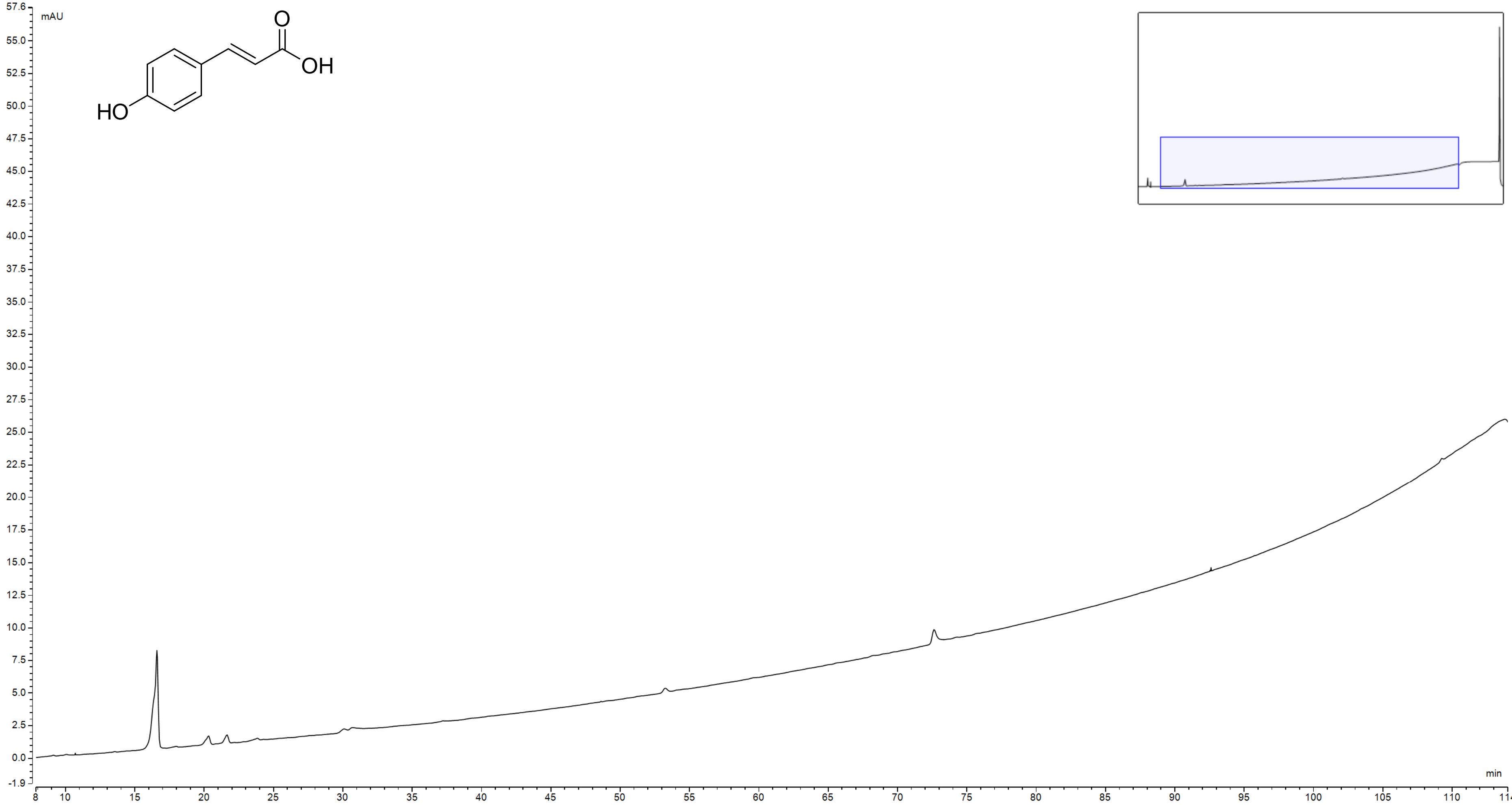

Compond **8**: 4-hydroxybenzaldehyde

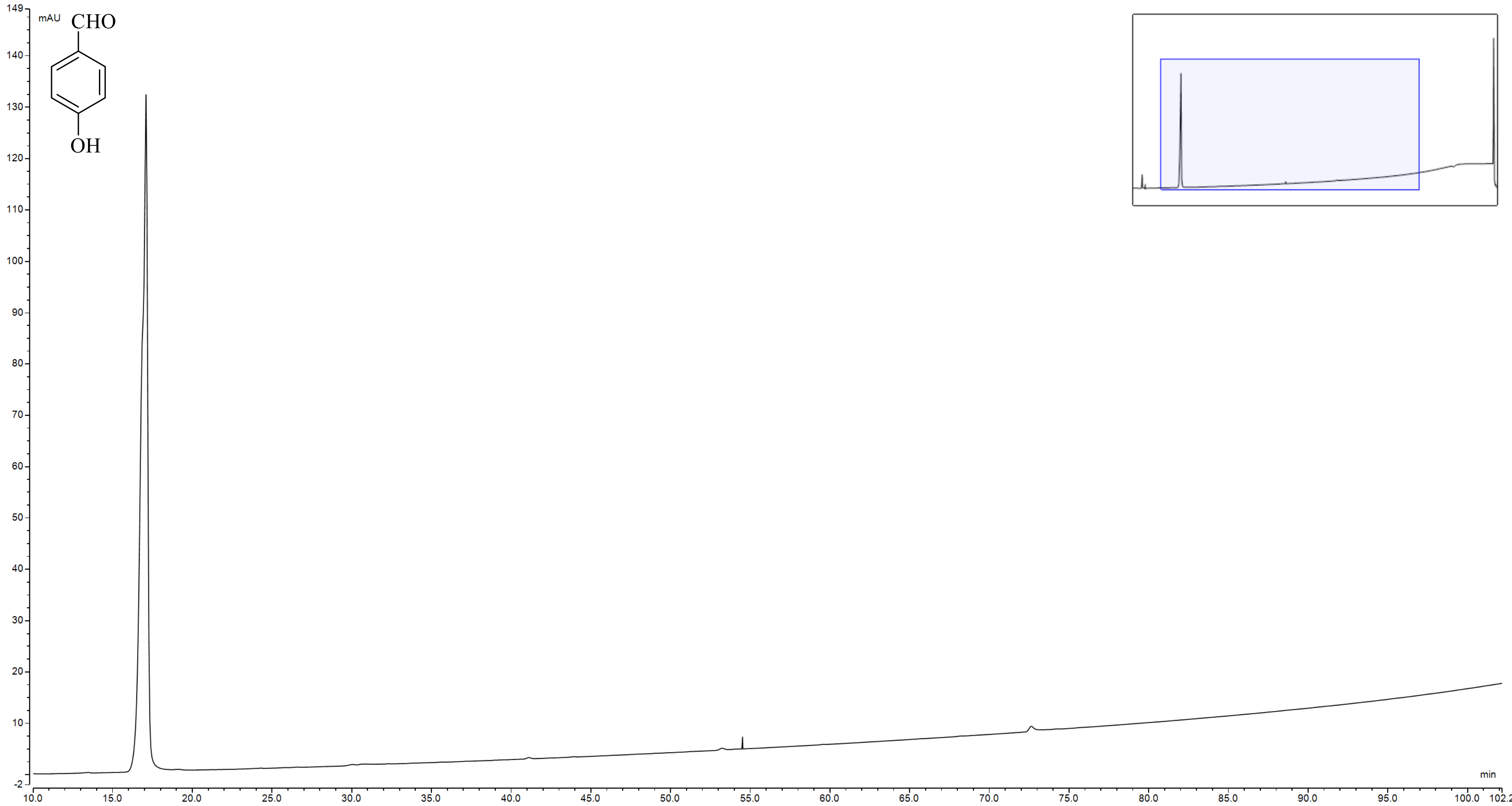

Compond **9**: vanillin

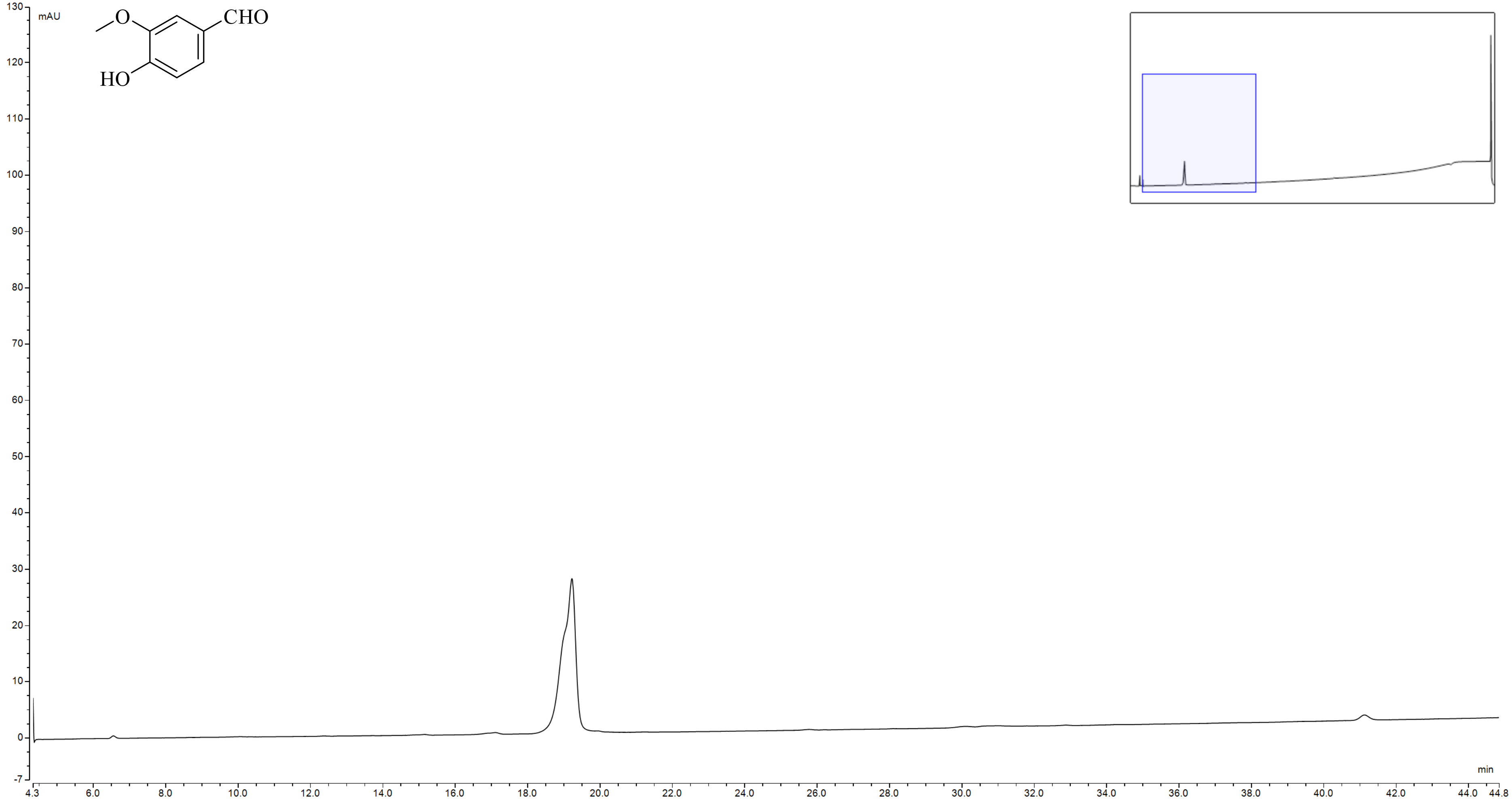

Compond **10**: 4-hydroxyacetophenone

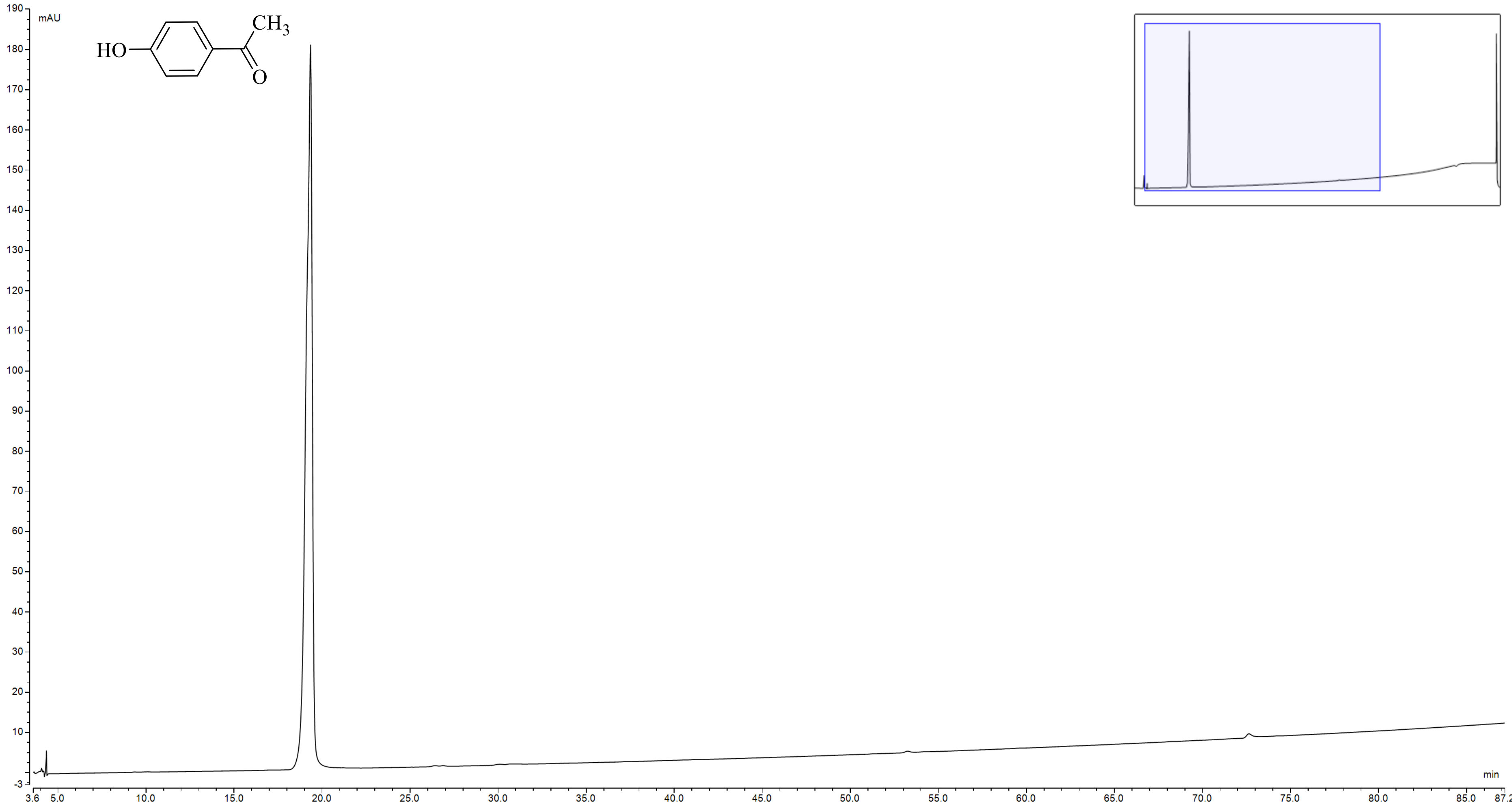

Compond **11**: 2,6-Dimethoxy-benzoic acid

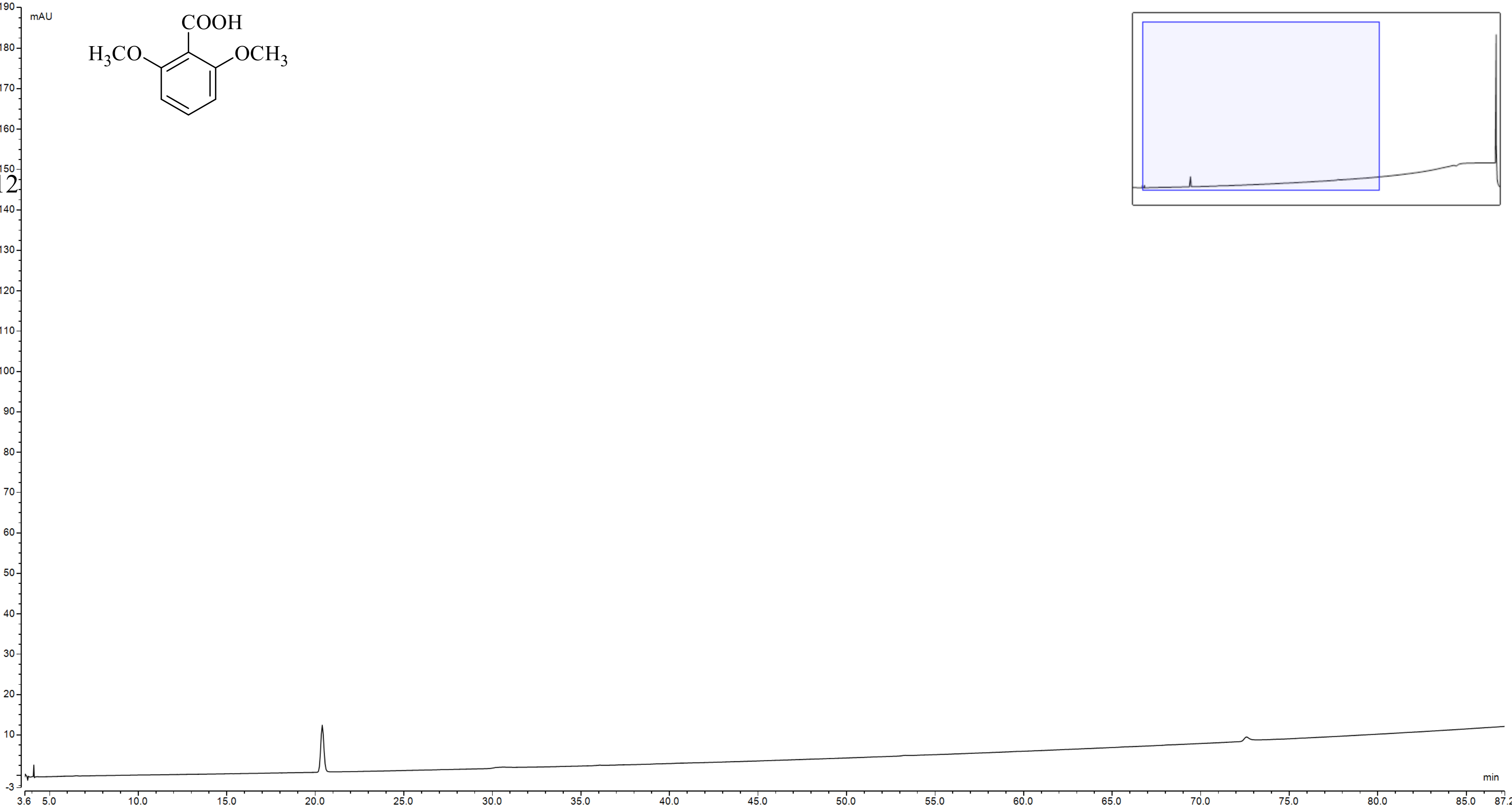

Compond **12**: coniferyl aldehyde

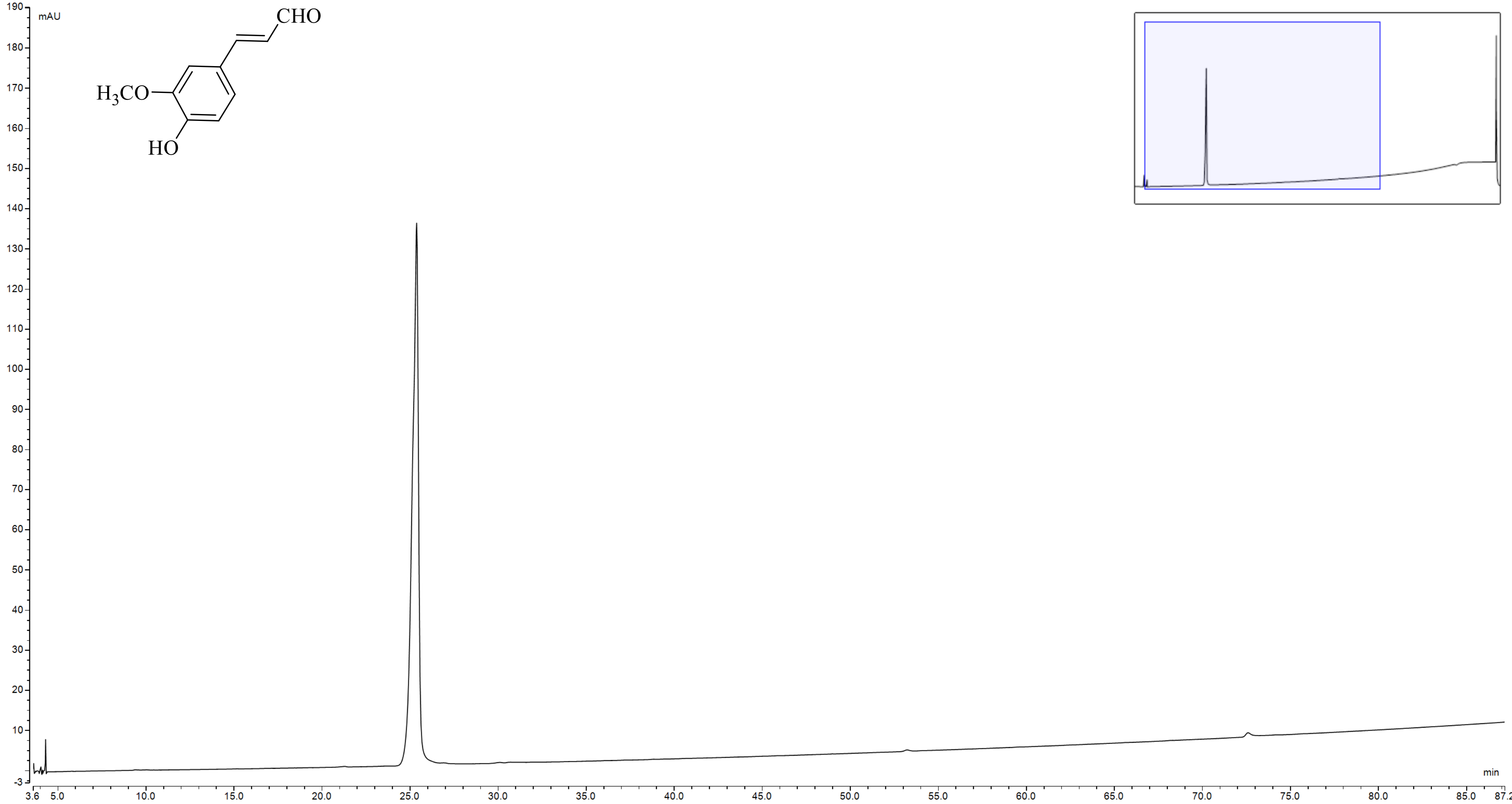

Compond **13**: ethyl shikimate

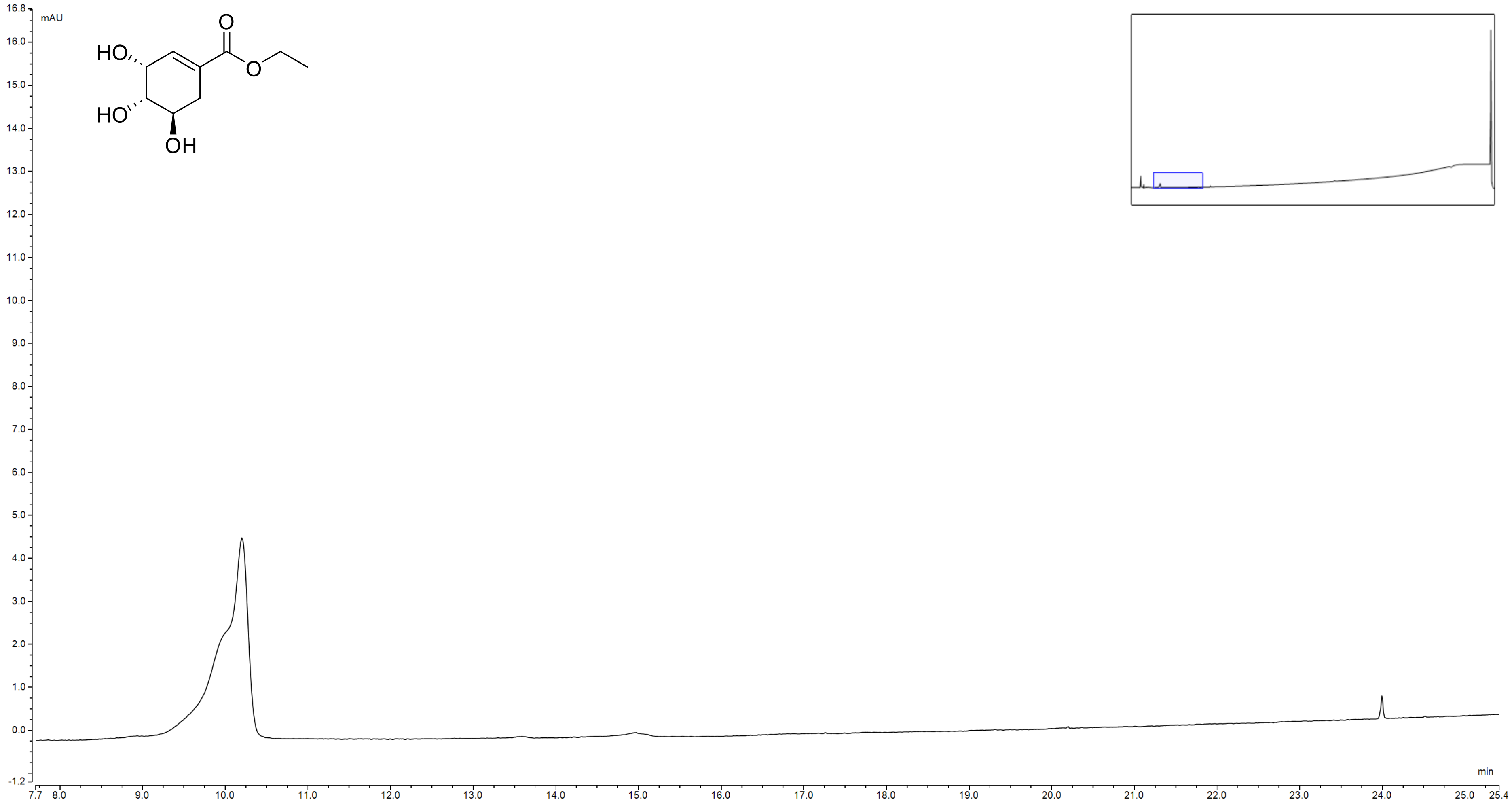

Compond **14**: sinensigenin C

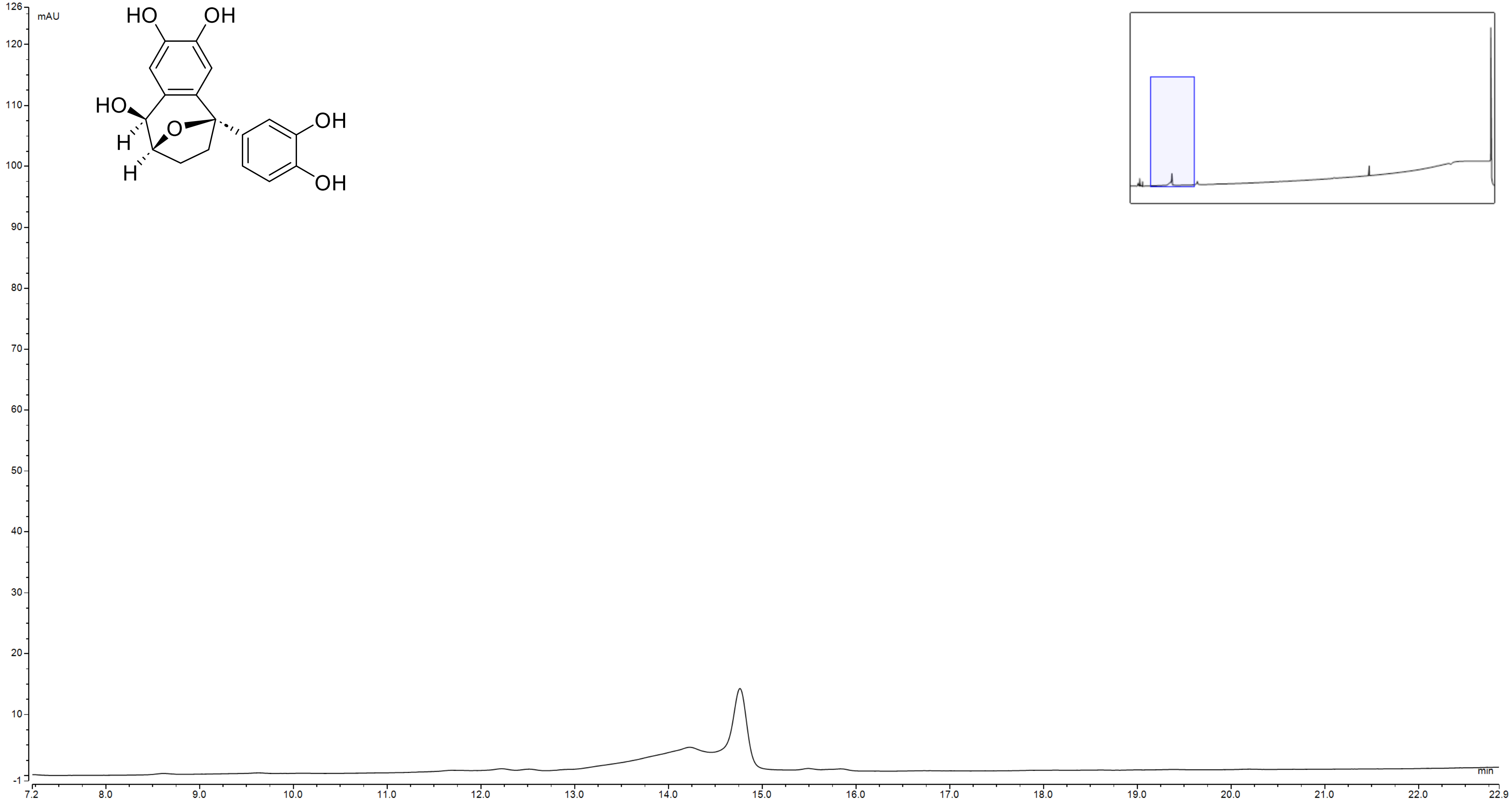

Compond **15**: erythro-guaiacylglycerol 8'-vanillic acid ether

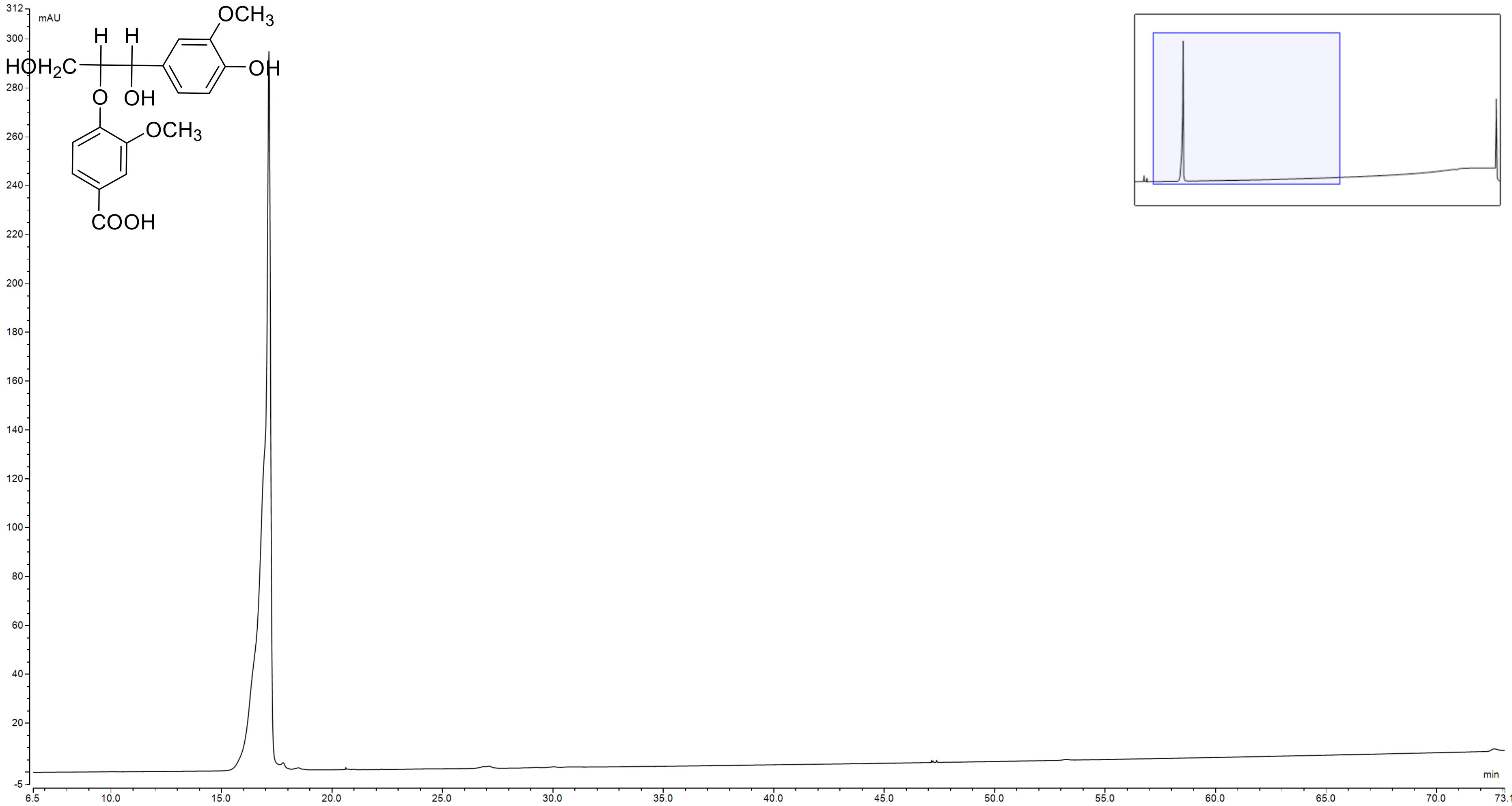

Compond **16**: crassifogenin B

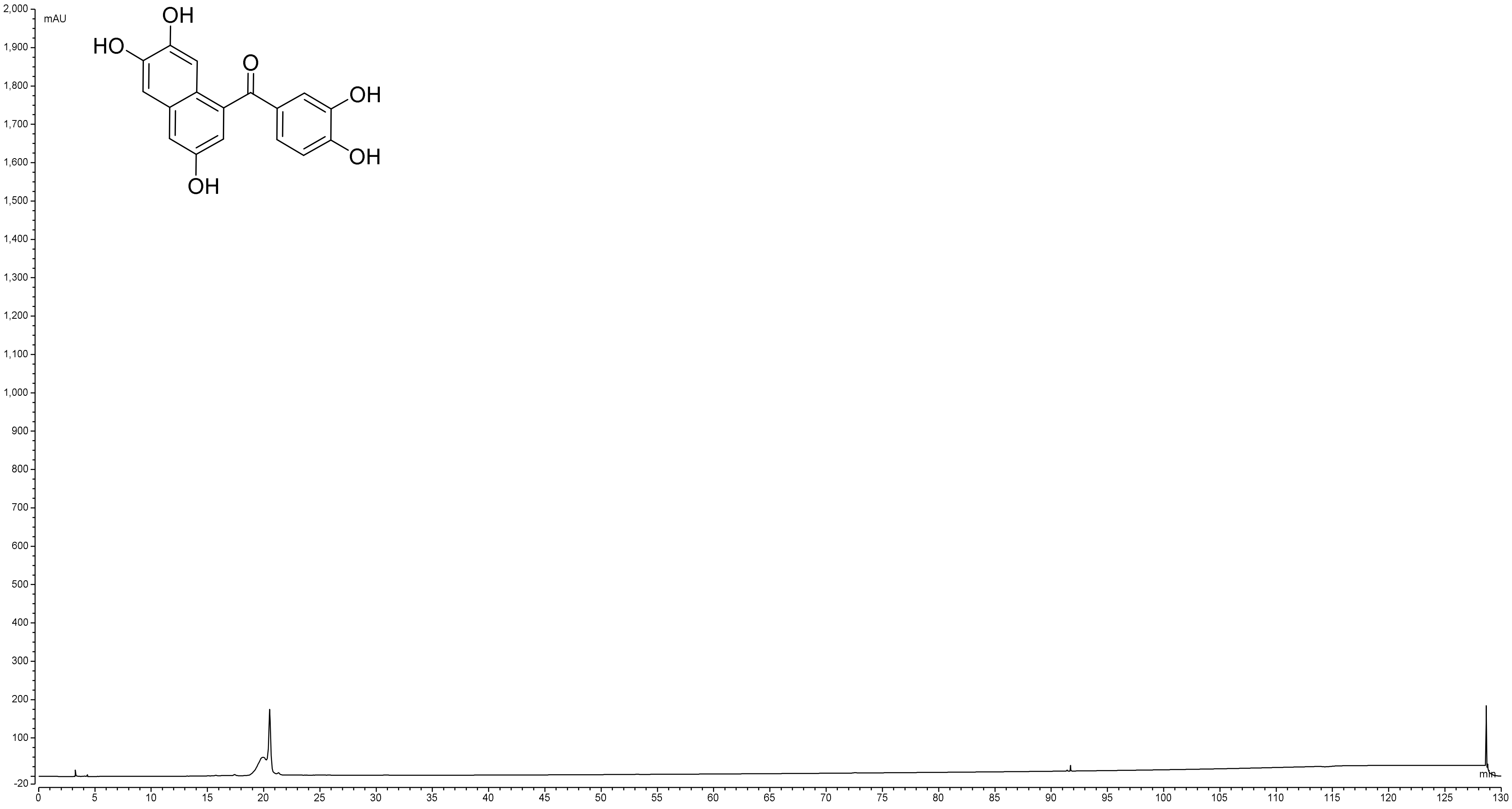

Compond **17**: curculigoside I

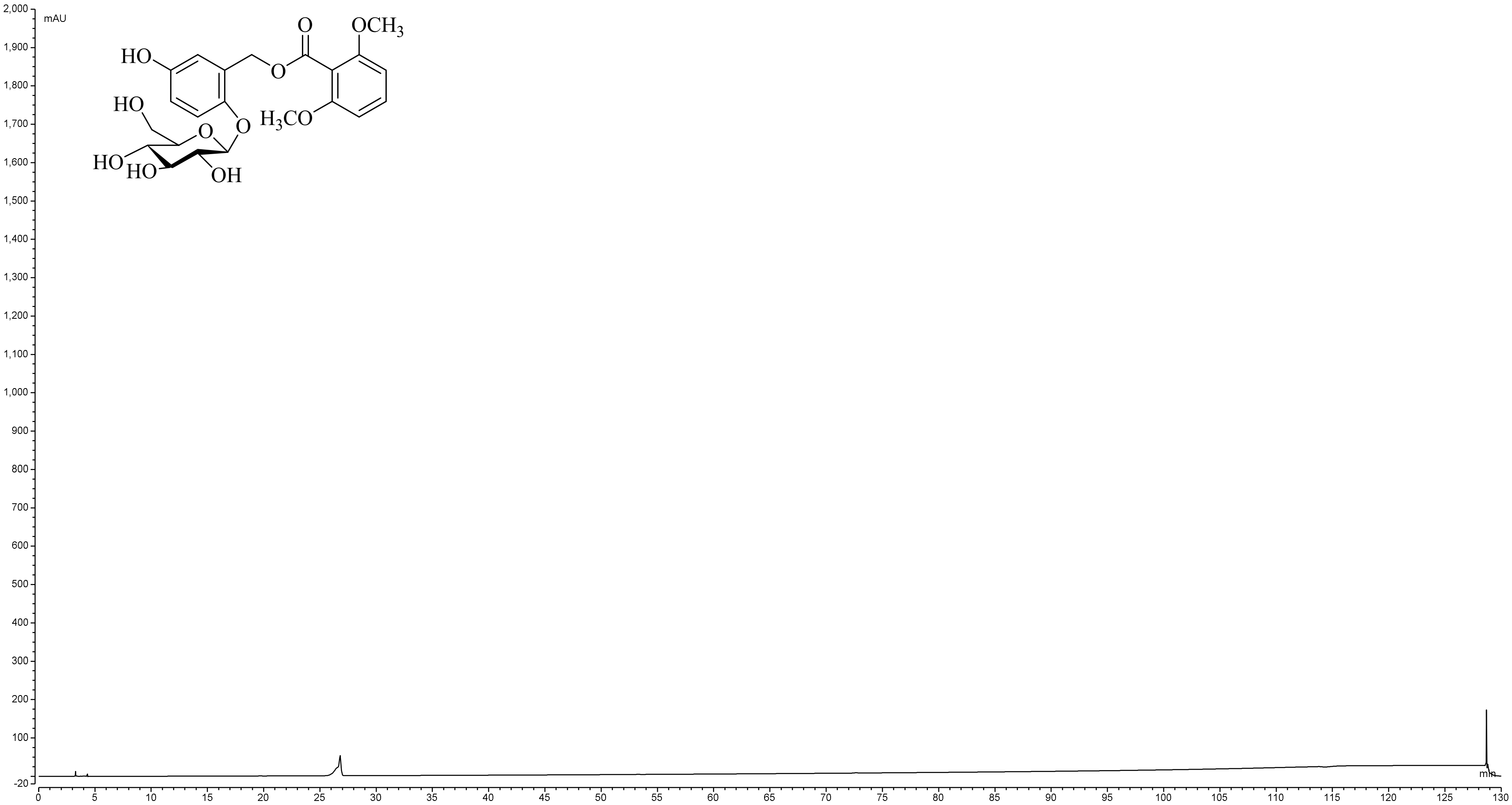

Compond **18**: 3,4-dihydroxyphenylethyl alcohol

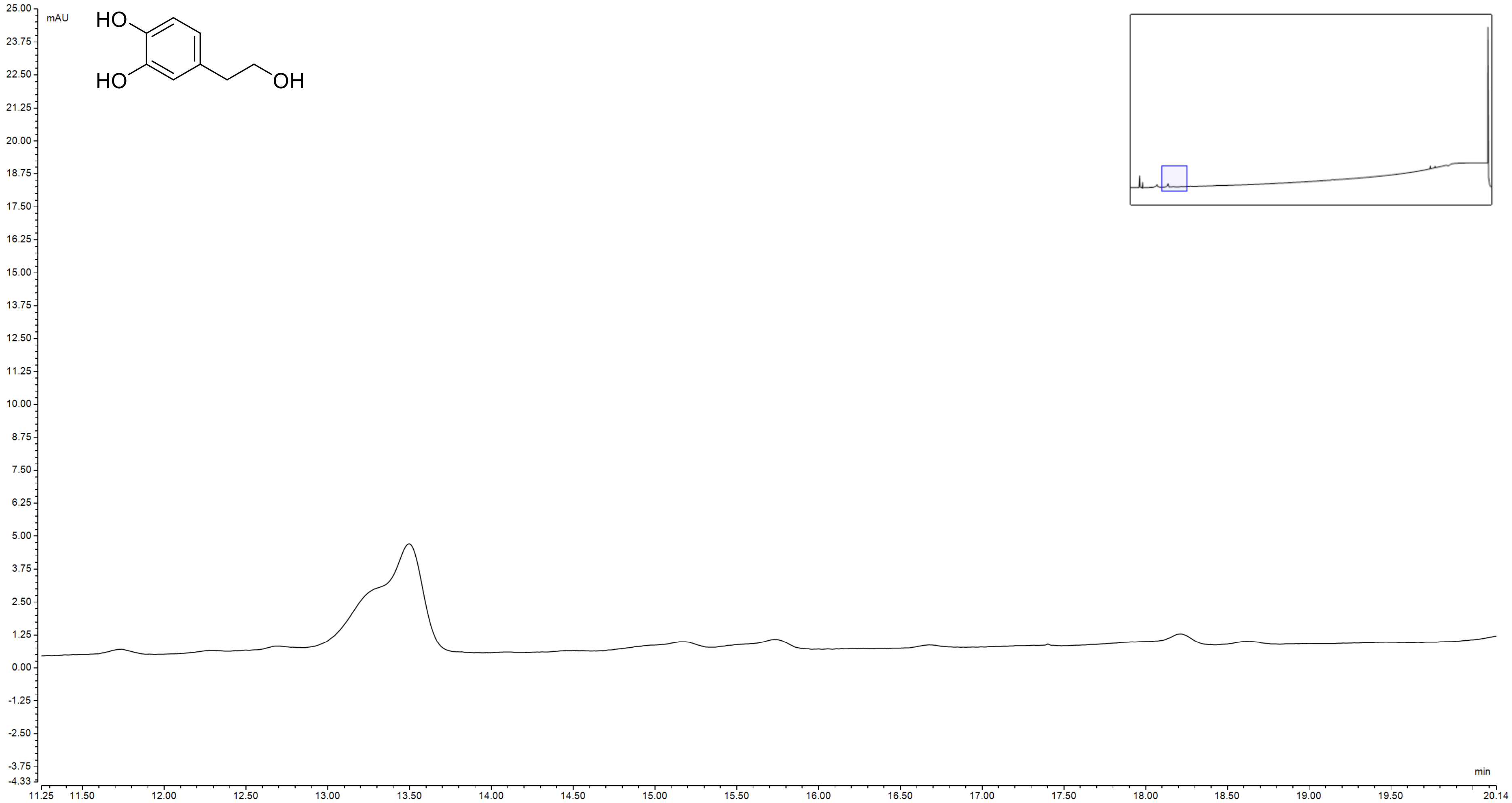

Compond **19**: threo-5-hydroxy-3,7 dimethoxyphenylpropane-8,9-diol

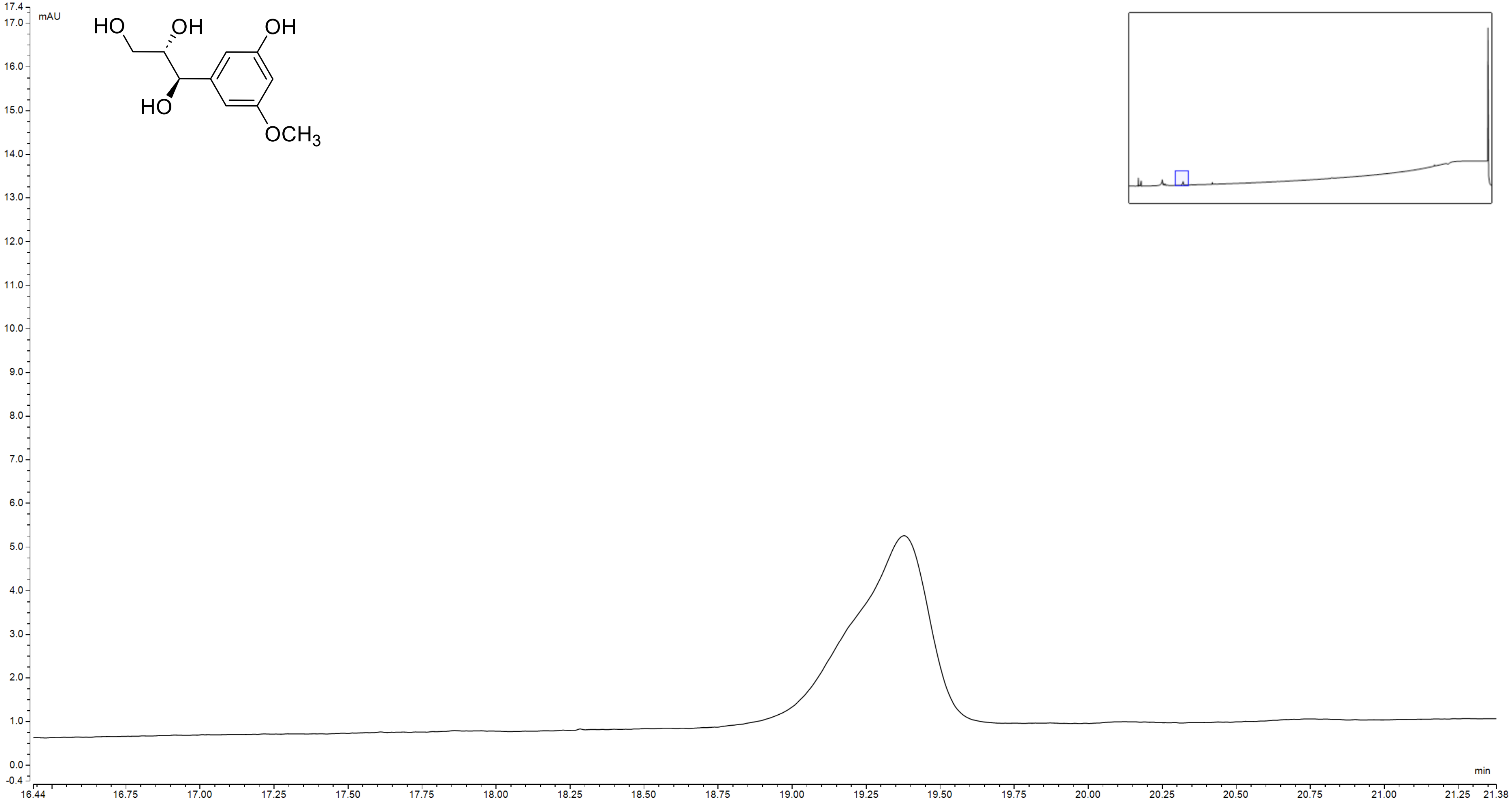

Compond **20**: threo-guaiacylglycerol 8'-vanillic acid ether

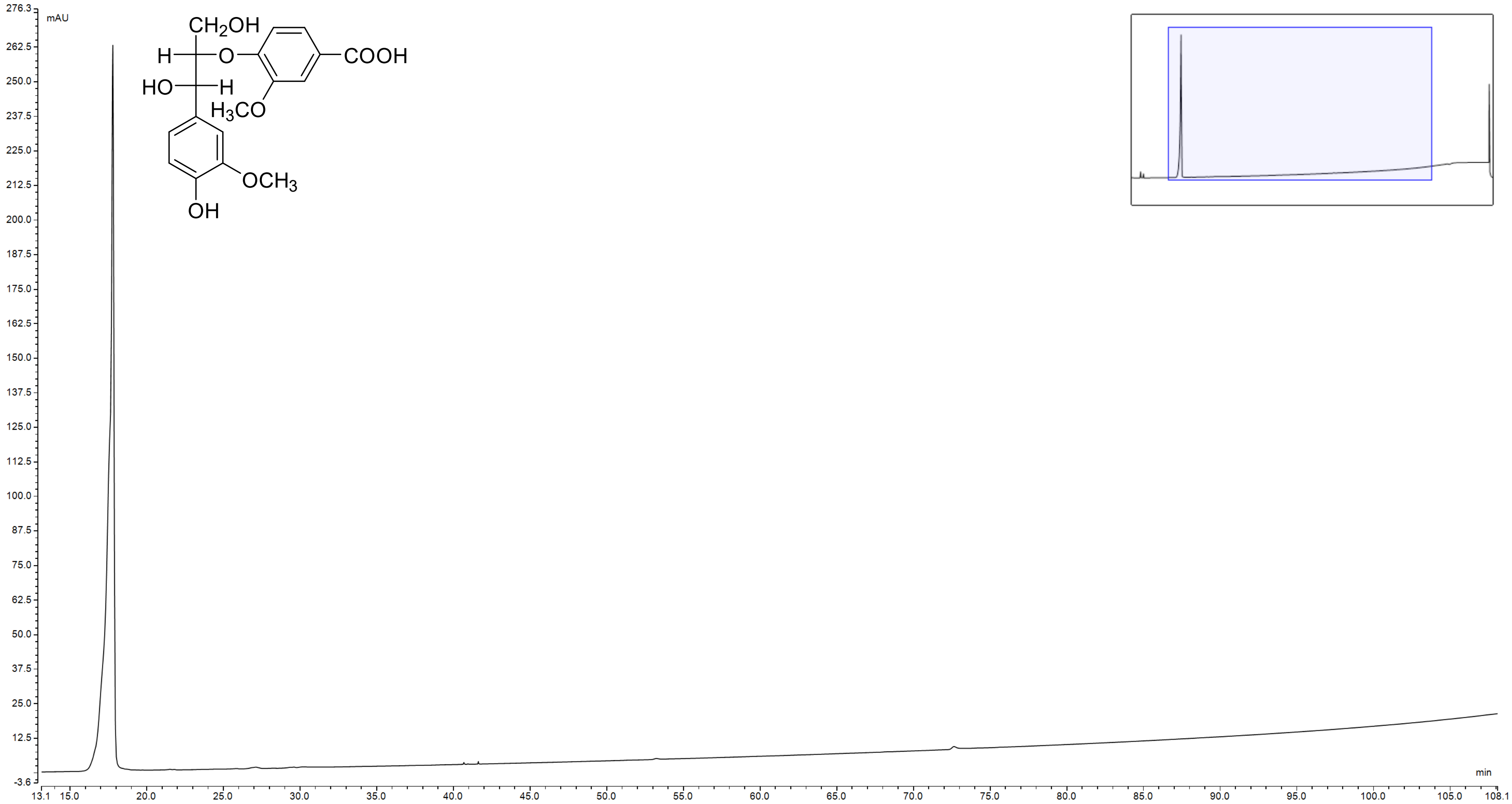

Compond **21**: (1R, 2R)-crassifogenin D

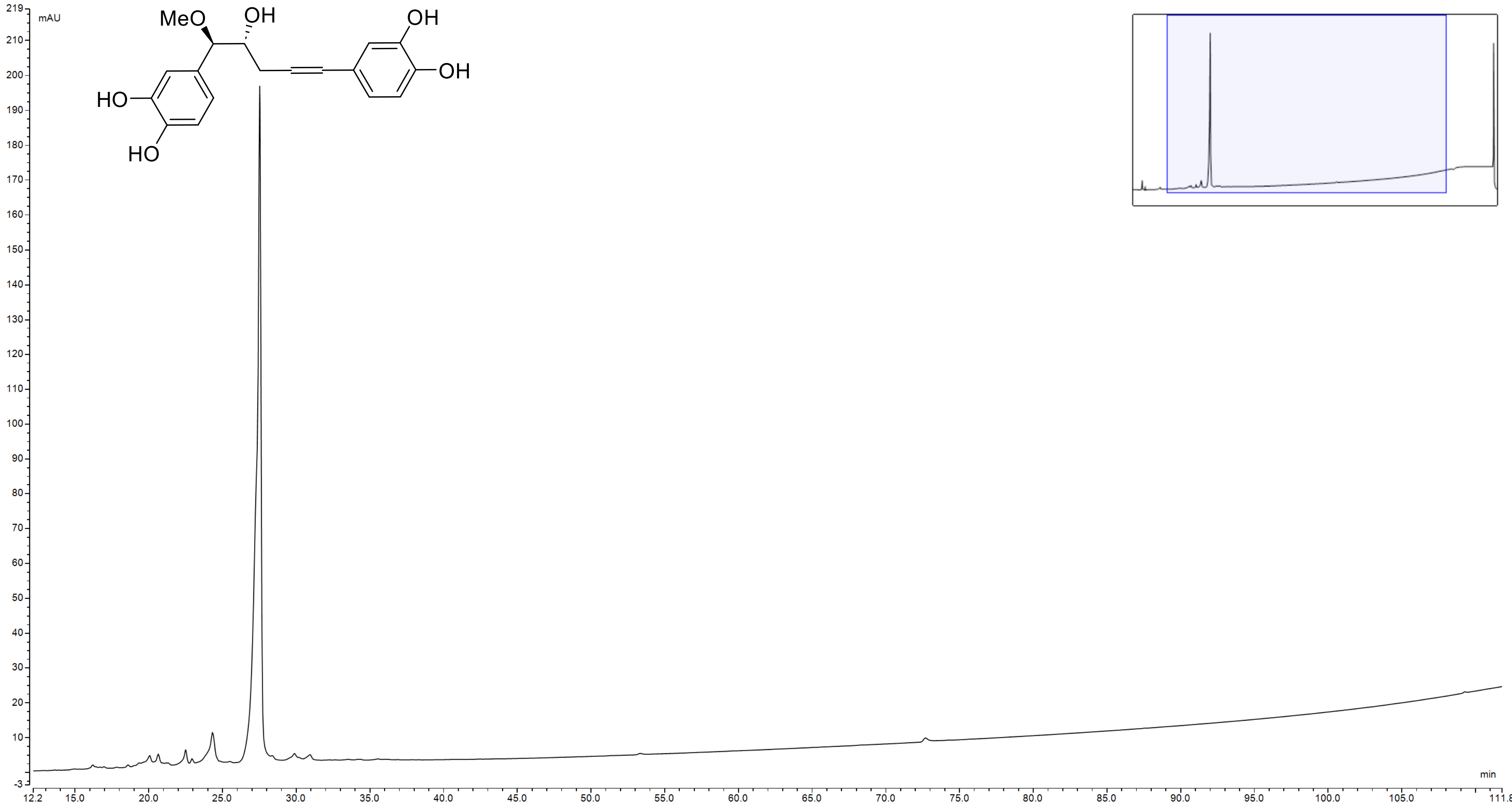

Compond **22**: 4-ketopinoresinol

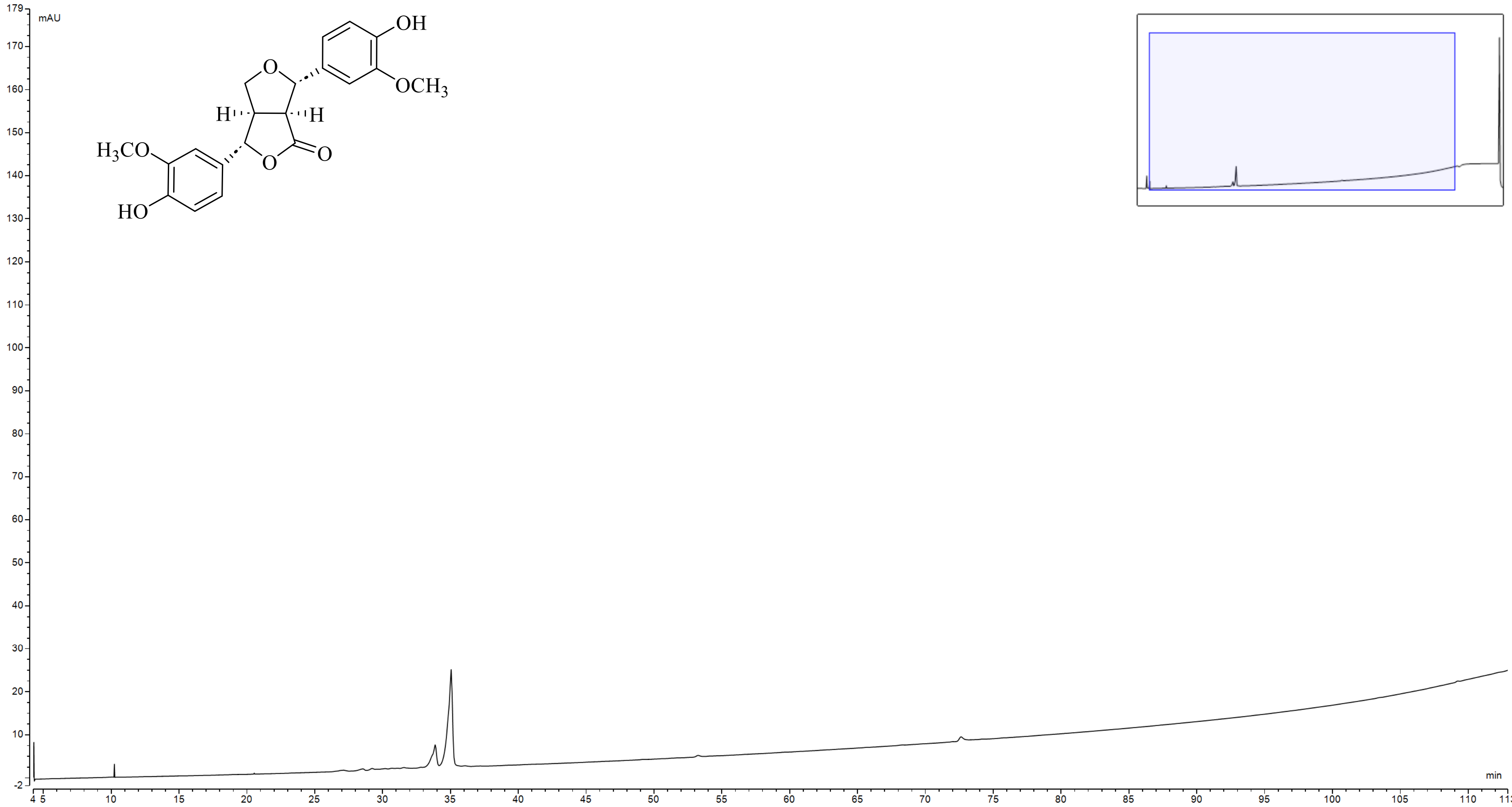

Supplement: Supplementary file 1 [file cells-13-02028-s001.zip › Standard Spectrum.pdf]
