# Supplementary material for: Extract of Curculigo capitulata Ameliorates Postmenopausal Osteoporosis by Promoting Osteoblast Proliferation and Differentiation
Source: Cells. 2024 Dec 8;13(23):2028. doi: 10.3390/cells13232028 (PMC11640542; doi:10.3390/cells13232028)
Supplement: Supplementary file 1 [file cells-13-02028-s001.zip › Table S1.pdf]

Table S1. The primers information of genes in this study.

Primer Name	Primer Sequence (5'to3')
Bglap-F	GCAGGAGGGCAATAAGGT
Bglap-R	CATAGATGCGTTTGTAGGC
Spp1-F	ACTCCAATCGTCCCTACA
Spp1-R	AGACTCACCGCTCTTCAT
Runx2-F	CCAACTTCCTGTGCTCCGTG
Runx2-R	TCTTGCCTCGTCCGCTCC
ibsp-F	CACCGCCCGAAGCCTAT
ibsp-R	CTCCCCGTTCTCGTTGTCA
GAPDH-F	GTGTTCCTACCCCCAATGTGTC
GAPDH-R	AGCCCAAGATGCCCTTCAGT
