# Supplementary material for: Extract of Curculigo capitulata Ameliorates Postmenopausal Osteoporosis by Promoting Osteoblast Proliferation and Differentiation
Source: Cells. 2024 Dec 8;13(23):2028. doi: 10.3390/cells13232028 (PMC11640542; doi:10.3390/cells13232028)
Supplement: Supplementary file 1 [file cells-13-02028-s001.zip › Table S2.pdf]

Table S2. Detailed information on the 22 components in Eocc.

Number	Name	Molecular weight	Molecular formula	retention time (min)	relative peak area (%)	Concentration (μg/ml)
**1**	acetovanillone	166.06	$C_9H_{10}O_3$	21.06	0.58	58
**2**	3,4-dihydroxyben zoic acid	154.02	$C_7H_6O_4$	9.6	0.52	52
**3**	gentisyl alcohol	140.04	$C_7H_8O_3$	9.043	0.25	25
**4**	2,6-dimethoxy-p-benzoquinone	168.04	$C_8H_8O_4$	14.83	0.77	77
**5**	vanillic acid	168.04	$C_8H_8O_4$	16.08	1.47	147
**6**	syringic acid	154.02	$C_7H_6O_4$	15.797	8.11	811
**7**	p-coumaric acid	164.04	$C_9H_8O_3$	16.517	1.68	168
**8**	4-hydroxybenzald ehyde	122.03	$C_7H_6O_2$	17.143	0.59	59
**9**	vanillin	152.04	$C_8H_8O_3$	18.843	0.51	51
**10**	4-hydroxyacetoph enone	136.05	$C_8H_8O_2$	19.207	0.21	21
**11**	2,6-Dimethoxy-b enzoic acid	182.05	$C_9H_{10}O_4$	20.343	0.21	21
**12**	coniferyl aldehyde	178.06	$C_{10}H_{10}O_3$	25.26	0.44	44
**13**	ethyl shikimate	202.08	$C_9H_{14}O_5$	10.077	0.24	24
**14**	sinensigenin C	316.09	$C_{17}H_{16}O_6$	14.71	0.54	54
**15**	erythro-guaiacylg lycerol 8'-vanillic acid ether	364.11	$C_{18}H_{20}O_8$	16.763	0.56	56
**16**	crassifogenin B	312.06	$C_{17}H_{12}O_6$	19.853	0.26	26
**17**	curculigoside I	466.14	$C_{22}H_{26}O_{11}$	26.74	0.13	13
**18**	3,4-dihydroxyphe nylethyl alcohol	154.06	$C_8H_{10}O_3$	13.417	1.59	159
**19**	threo-5-hydroxy-3,7 dimethoxyphenyl propane-8,9-diol	214.08	$C_{10}H_{14}O_8$	19.517	0.54	54
**20**	threo-guaiacylgly cerol 8'-vanillic acid ether	364.11	$C_{18}H_{20}O_8$	17.877	0.52	52
**21**	(1R, 2R)-crassifogenin D	332.12	$C_{18}H_{20}O_6$	27.483	1.01	101
**22**	4-ketopinoresinol	372.12	$C_{20}H_{20}O_7$	35.18	0.13	13
